# Psilocybin and Eugenol Reduce Inflammation in Human 3D EpiIntestinal Tissue

**DOI:** 10.3390/life13122345

**Published:** 2023-12-15

**Authors:** Gregory Ian Robinson, Dongping Li, Bo Wang, Tahiat Rahman, Marta Gerasymchuk, Darryl Hudson, Olga Kovalchuk, Igor Kovalchuk

**Affiliations:** 1 Department of Biological Sciences, University of Lethbridge, Lethbridge, AB T1K 3M4, Canada; 2 GoodCap Pharmaceuticals, 520 3rd Avenue SW, Suite 1900, Calgary, AB T2P 0R3, Canada

**Keywords:** psilocybin, ketanserin, 4-AcO-DMT, curcumin, eugenol, capsaicin, serotonin receptor ligands, transient receptor potential channel ligands, inflammation, small intestine, 3D tissue

## Abstract

Inflammation plays a pivotal role in the development and progression of inflammatory bowel disease (IBD), by contributing to tissue damage and exacerbating the immune response. The investigation of serotonin receptor 2A (5-HT2A) ligands and transient receptor potential (TRP) channel ligands is of significant interest due to their potential to modulate key inflammatory pathways, mitigate the pathological effects of inflammation, and offer new avenues for therapeutic interventions in IBD. This study investigates the anti-inflammatory effects of 5-HT2A ligands, including psilocybin, 4-AcO-DMT, and ketanserin, in combination with TRP channel ligands, including capsaicin, curcumin, and eugenol, on the inflammatory response induced by tumor necrosis factor (TNF)-α and interferon (IFN)-γ in human 3D EpiIntestinal tissue. Enzyme-linked immunosorbent assay was used to assess the expression of pro-inflammatory markers TNF-α, IFN-γ, IL-6, IL-8, MCP-1, and GM-CSF. Our results show that psilocybin, 4-AcO-DMT, and eugenol significantly reduce TNF-α and IFN-γ levels, while capsaicin and curcumin decrease these markers to a lesser extent. Psilocybin effectively lowers IL-6 and IL-8 levels, but curcumin, capsaicin, and 4-AcO-DMT have limited effects on these markers. In addition, psilocybin can significantly decrease MCP-1 and GM-CSF levels. While ketanserin lowers IL-6 and GM-CSF levels, there are no effects seen on TNF-α, IFN-γ, IL-8, or MCP-1. Although synergistic effects between 5-HT2A and TRP channel ligands are minimal in this study, the results provide further evidence of the anti-inflammatory effects of psilocybin and eugenol. Further research is needed to understand the mechanisms of action and the feasibility of using these compounds as anti-inflammatory therapies for conditions like IBD.

## 1. Introduction

The intestinal epithelium acts as a permeable and dynamic interface for the absorption of water, dietary nutrients, and electrolytes, while maintaining an important barrier to prevent the entry of pathogenic microorganisms found in the gut [1,2]. While the intestinal mucosa prevents the translocation of toxins, microorganisms, and antigens, inflammation plays a vital role by eliminating these harmful agents. However, excessive intestinal inflammation can lead to inflammatory bowel disease (IBD), and secondary extraintestinal manifestations, including diseases in the hepatobiliary, musculoskeletal, dermatological, renal, and pulmonary systems [3]. While the triggers of IBD are multifactorial and complex, inflammation plays a critical role in the pathogenesis of IBD [4].

Due to the large global burden with over 4.9 million global cases of IBD [5] and rising prevalence rates [6], there is a growing need for novel therapeutics to treat IBD [4]. The therapeutic potential of currently approved drugs are limited and controversial due to limited efficacy, poor safety and tolerability profiles, or adverse side effects [4].

Recently, an increasing amount of research is looking into the anti-inflammatory potential of psychedelic mushrooms. Extracts of psychedelic mushrooms have been shown to decrease nitrosative stress and the production of inflammatory cytokines and prostaglandins in lipopolysaccharide (LPS)-activated mouse and human macrophages in vitro [7,8]. Since psychedelic mushrooms contain psilocybin, many have presumed the anti-inflammatory effects were due to the binding of psilocybin’s active metabolite psilocin on the 5-HT2A receptor, which is known to modulate immune function and cytokine production [9]. Although psilocin can bind to 5-HT2B receptors [10], pivotal studies show the anti-inflammatory effects of psychedelics are mediated by the 5-HT2A receptor. Furthermore, psilocybin has been shown to have superior anti-inflammatory potential compared to non-psychedelic 5-HT2A receptor agonists due to ligand specificity, and the anti-inflammatory effects of psilocybin do not require hallucinogenic doses in animal models [9].

Other novel psychedelics have been suggested as potential anti-inflammatory therapeutics, including a synthetic analogue of psilocybin, 4-acetoxy-N,N-dimethyltryptamine (4-AcO-DMT), which is the O-acetylated version of psilocin and is deacylated within the liver to produce psilocin. Due to lower production costs and relative ease to synthesize 4-AcO-DMT, it has been suggested as a replacement for psilocybin [11]; however, there has been no published research on the anti-inflammatory effects of 4-AcO-DMT until recently [12].

Ketanserin, though tethered to a reputation marked by adverse effects, has shown unexpected anti-inflammatory capabilities. Despite being an antagonist of the 5-HT2A receptor, ketanserin has been shown to alleviate colitis by preventing neutrophil infiltration, inflammatory cytokine production, and cellular apoptosis [13]. Simultaneously, ketanserin can curtail M2 macrophage polarization, migration, and NF-κB activation, all while restoring the intestinal mucosa’s architectural integrity [14]. While ketanserin has shown potential in inhibiting inflammation in IBD, ketanserin has known adverse effects that would likely contraindicate any therapeutic use in IBD, including causing cardiac arrythmias [15], dyspepsia [16], and orthostatic hypotension [17].

In addition, many researchers are studying transient receptor potential channels (TRP) due to their presence on monocytes/macrophages [18], as well as recent evidence showing their importance in inflammation and inflammatory diseases [19]. TRP vanilloid 1 (TRPV1) and TRP melastatin 8 (TRPM8) have recently been shown to modulate LPS-induced inflammation [20] while binding can prevent inflammation and oxidative stress [21]. As such, we have chosen to study TRP ligands eugenol, curcumin, and capsaicin.

Previously, we have shown eugenol has potent anti-inflammatory effects and shows synergy with psilocybin in vivo within the brain [22], as well as in vitro within small intestinal epithelial cells [12]; however, outside of cell models, the effects of eugenol on IBD still remains unknown. Although eugenol is still being studied, it is demonstrating potential as an IBD therapeutic and has been declared by World Health Organization (WHO) to be generally recognized as safe and non-mutagenic [23].

In contrast, curcumin, found in turmeric, has potent anti-inflammatory effects, specifically inhibiting toll-like receptor 4 (TLR4)/NF-κB-induced inflammation [24] and by binding to TRPV1 to prevent IL-6 and TNF-α production [25]. Curcumin has been suggested as a potential remedy for IBD due to an acceptable daily intake of up to 3 mg/kg of the body weight, as recognized by the Food Agriculture Organization, WHO, and European Food Safety Authority [26].

In addition, capsaicin can activate TRPV1 to inhibit NF-κB signaling and production of pro-inflammatory cytokines and COX-2 [27,28,29]. Furthermore, capsaicin has been shown to decrease high fat diet-induced endotoxemia by preventing microbial dysbiosis, gut barrier dysfunction, and low-grade inflammation [30]. While capsaicin does have beneficial anti-inflammatory effects, its use is limited due to an estimated LD_50_ of 5–50 g/kg of the body weight, and unpleasant side effects at doses well below these levels, including only 10 mg required to induce intestinal cramping and discomfort [31].

Our study aims to investigate the efficacy of 5-HT2A ligands, including psilocybin, 4-AcO-DMT, and ketanserin, as well as TRP channel ligands, including capsaicin, curcumin, and eugenol, on the inflammatory response to TNF-α/INF-γ in human 3D EpiIntestinal tissue. In addition, the synergism between drug classes were tested to determine whether synergistic anti-inflammatory effects previously seen in cellular models [12] could be replicated within this 3D tissue model of inflammation. Due to biased agonism and ligand specificity [32], we hypothesized that psilocybin would prove to be the most efficacious single treatment and could provide synergistic benefits when paired with eugenol. The results of this study will expand our understanding on the purpose of 5-HT2A and TRP ligands in inflammation and provide guidance for designing novel anti-inflammatory therapeutics.

## 2. Materials and Methods

### 2.1. EpiIntestinal 3D Model and Inflammation Induction

An EpiIntestinal tissue model (SMI-100-FT-HCF ver 2.0, MatTek, Ashland, MA, USA) was used due to the 3D composition mimics the structure of brush borders, functional tight junctions, and mucous-secreting granules, similar to in vivo human small intestines. The 3D reconstructed tissue model contains primary, human cell-derived small intestine epithelial, endothelial cells and fibroblasts. It exhibits *in vivo*-like growth and morphological characteristics, whereby cells sustain differentiation and metabolic status similar to those of human intestinal epithelium [33].

**Figure 1:**
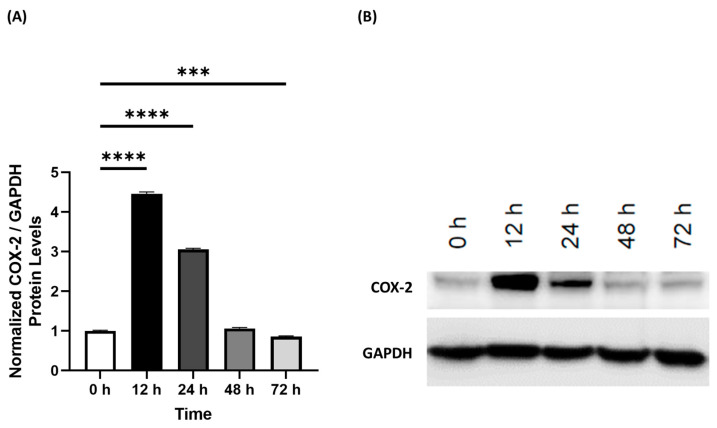
Time-course of COX-2 protein levels from 0 to 72 h after induction of an inflammatory response by treatment with TNF-α/IFN-γ in human 3D EpiIntestinal tissue. (**A**) Normalized densitometry of COX-2 protein levels compared to GAPDH. (**B**) Membranes of COX-2 and GAPDH. Original blots can be found in Figures S1 and S2 within the supplementary materials. Bars represent the mean ± SD. Data were analyzed with a one-way ANOVA test and a Dunnett’s post hoc multiple comparison test compared to the 0 h group. Significance is indicated within the figures using the following scale: *** *p* < 0.001, **** *p* < 0.0001.

To establish the TFN-α/IFN-γ induction of inflammation, 3D EpiIntestinal tissue was cultured according to the manufacturer’s instructions. Upon arrival, the 3D tissue was received and stored at 4 °C. On the same day as arrival, the 3D EpiIntestinal tissue was incubated in 5.0 mL of maintenance medium (SMI-100-MM, MatTek) in 6-well plates at a humidified atmosphere of 5% CO_2_. After 24 h of incubation, the EpiIntestinal 3D tissues were treated with 10 ng/mL TFN-α/IFN-γ (Sigma, Markham, ON, Canada) and dissolved in the maintenance media for 12 h. The protein levels of COX-2 (Figure S1) and GAPDH (Figure S2) were measured via Western blot to confirm the induction of inflammation and can be seen in Figure 1.

### 2.2. Treatment of EpiIntestinal 3D Tissue

To determine the anti-inflammatory properties of psilocybin (Sigma, Markham, ON, Canada), 4-AcO-DMT (ChemLogix, Burnaby, BC, Canada), ketanserin (TCI America, Portland, OR, USA), capsaicin (Sigma, Markham, ON, Canada), curcumin (Sigma, Markham, ON, Canada), and eugenol (Sigma, Markham, ON, Canada), EpiIntestinal 3D tissues were treated with 10 ng/mL TFN-α/IFN-γ alone or in combination with the indicated concentration of psilocybin, 4-AcO-DMT, ketanserin, capsaicin, curcumin, and eugenol either individually or in combination for 12 h. The doses utilized for psilocybin include: 0, 10, 20, and 40 μM. The doses utilized for 4-AcO-DMT include: 0, 20, and 40 μM. The doses used for ketanserin include: 0, 1, 5, and 10 μM. The doses used for curcumin include: 0, and 0.5 μM. The doses used for capsaicin include: 0 and 0.5 μM. The doses used for eugenol include: 0 and 25 μM.

### 2.3. Multiplex Enzyme-Linked Immunosorbent Assay (ELISA)

The media in which the tissues were grown in were analyzed. Samples were snap-frozen in liquid nitrogen and stored at −80 °C until utilized. Samples were submitted to and processed by Eve technologies (Calgary, AB, Canada) for testing. Among all cytokines analyzed, the following gave detectable signal GM-CSF, IFN-γ, IL-6, IL-8, MCP-1, and TNF-α within the detection range.

Eve Technologies Corp. (Calgary, AB, Canada) used Luminex xMAP technology for multiplexed quantification of 13 human cytokines, chemokines, and growth factors. The multiplexing analysis was performed using the Luminex™ 200 system (Luminex, Austin, TX, USA). Thirteen markers were simultaneously measured in the samples using Eve Technologies’ Human Focused 13-Plex Discovery Assay^®^ (MilliporeSigma, Burlington, MA, USA), according to the manufacturer’s protocol. The 13-plex consisted of GM-CSF, IFNγ, IL-1β, IL-2, IL-4, IL-5, IL-6, IL-8, IL-10, IL-12p70, IL-13, MCP-1, and TNF-α. The assay sensitivities of these markers ranged from 0.14 to 5.39 pg/mL for the 13-plex. Individual analyte sensitivity values are available in the MilliporeSigma MILLIPLEX^®^ MAP protocol.

### 2.4. Whole Cellular Lysate Preparation and Western Blot Analysis

Three-dimensional tissue membranes were cut off with a surgical blade, placed in a 1.7 mL microtube containing 30 µL RIPA, and immersed in liquid nitrogen immediately. The whole cellular lysates of 3D tissues were prepared in RIPA buffer using 2.0 mm ZR BashingBead beads (Zymo Research, Irvine, CA, USA). Lysates were centrifuged at 12,000× *g* for 10 min. The supernatant was collected and stored at −80 °C until further use.

To quantify protein concentrations, the Bradford protein assay was performed via NanoDrop 2000/2000c Spectrophotometer (Thermo Fisher Scientific, Wilmington, DE, USA). Between 60 and 100 μg of protein per sample was electrophoresed on 8% or 10% SDS-PAGE and electrophoretically transferred to a PVDF membrane (Amersham Hybond™-P, GE Healthcare, Chicago, IL, USA) at 4 °C for 1.5 h. Blots were incubated for 1 h with 5% non-fat dry milk to block nonspecific binding sites and subsequently incubated at 4 °C overnight with a 1:1000 dilution of polyclonal antibody against COX-2 (Abcam, Cambridge, UK). Immunoreactivity was detected using a peroxidase-conjugated antibody and visualized by an ECL Plus Western Blotting Detection System (GE Healthcare, Chicago, IL, USA). The blots were stripped before re-probing with antibody against GAPDH (Abcam, Cambridge, UK). The densitometry of bands was measured and normalized with that of GAPDH using ImageJ.

### 2.5. Statistical Analysis

A one-way ANOVA followed by Dunnett’s post-hoc test was used to determine the statistical significance on all graphs with α = 0.05. Each experiment was performed a minimum of three times. Statistical analysis was performed on all samples that had a signal detected with a minimum of two samples. The results are represented by the mean and standard deviation (SD) of the mean. The mean and SD were calculated, analyzed, and plotted with GraphPad Prism 10.0.2 (GraphPad Software, San Diego, CA, USA). Fold changes were calculated by dividing the mean of the treatment by the mean of the control. Negative fold changes were calculated for all results less than 1. This was determined by taking the negative reciprocal of the fold change. Significance (*p*) was indicated within the figures using the following scale: * *p* < 0.05, ** *p* < 0.01, *** *p* < 0.001, and **** *p* < 0.0001.

## 3. Results

### 3.1. Human 3D EpiIntestinal Tissue Exhibits Inflammation Characteristic of Inflammatory Bowel Disease

Human 3D EpiIntestinal tissue was treated with TNF-α/IFN-γ to induce an inflammatory response. At 12, 24, 48, and 72 h, the normalized densitometry of COX-2 protein level was measured relative to GAPDH (Figure 1) to determine the timeline of the inflammatory response of the 3D tissue to TNF-α/IFN-γ. After 12 h, relative COX-2 protein level was significantly higher than the control (*p* < 0.0001) and had the highest levels out of any timepoint (Figure 1). In addition, relative COX-2 levels were also significantly upregulated at 24 h (*p* < 0.0001, Figure 1A). In contrast, relative COX-2 levels were unaltered after 48 h and were significantly lower at 72 h compared to the control (*p* < 0.001, Figure 1A). Representative membranes of each timepoint were imaged and displayed in Figure 1B.

### 3.2. Psilocybin and Transient Receptor Potential Channel Agonists Decrease Inflammation in Human 3D EpiIntestinal Tissue

**Figure 2:**
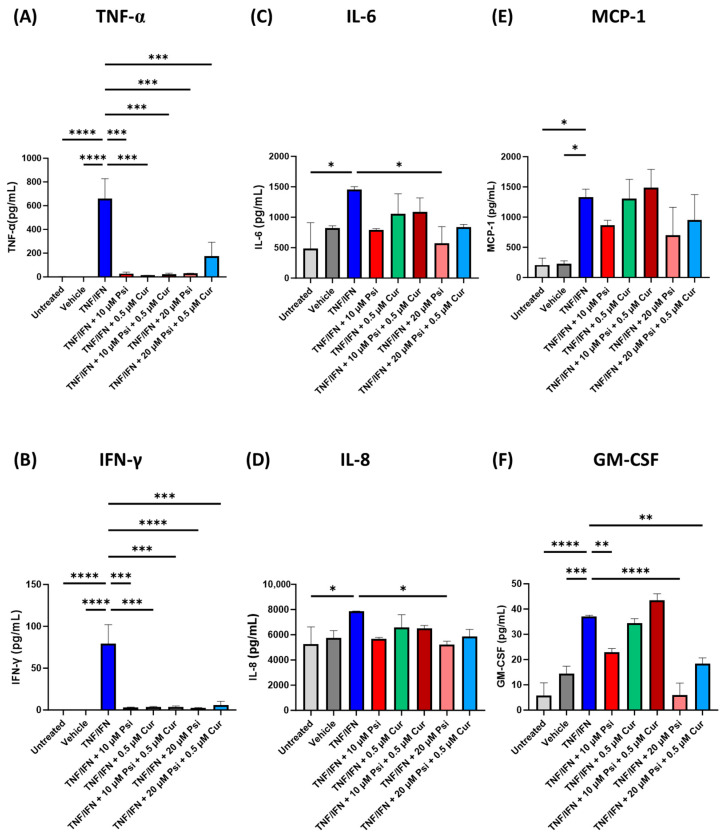
Psilocybin and curcumin combinations alter protein levels of inflammatory cytokines, chemoattractants, and growth factors in human 3D EpiIntestinal tissue. Protein levels measured via ELISA for (**A**) TNF-α, (**B**) IFN-γ, (**C**) IL-6, (**D**) IL-8, (**E**) MCP-1, and (**F**) GM-CSF. Bars represent the mean ± SD. Data were analyzed with a one-way ANOVA test and Dunnett’s post hoc multiple comparison test compared to the TFN-α/IFN-γ group. Significance is indicated within the figures using the following scale: * *p* < 0.05, ** *p* < 0.01, *** *p* < 0.001, **** *p* < 0.0001. Cur, curcumin; Psi, psilocybin.

A combination of psilocybin and curcumin were applied to human 3D EpiIntestinal tissue to test the synergistic effects between these compounds on the inflammatory response induced by TNF-α/IFN-γ. Compared to the untreated group, the levels of TNF-α (*p* < 0.001, Figure 2A), IFN-γ (*p* < 0.0001, Figure 2B), IL-6 (*p* < 0.05, Figure 2C), IL-8 (*p* < 0.05, Figure 2D), MCP-1 (*p* < 0.05, Figure 2E), and GM-CSF (*p* < 0.0001, Figure 2F) were significantly upregulated in the TNF-α/IFN-γ group. Similarly, TNF-α (*p* < 0.001, Figure 2A), IFN-γ (*p* < 0.0001, Figure 2B), MCP-1 (*p* < 0.05, Figure 2E), and GM-CSF (*p* < 0.001, Figure 2F) were significantly higher in the TNF-α/IFN-γ group compared to the vehicle.

All treatment groups significantly lowered TNF-α (*p* < 0.001, Figure 2A) and IFN-γ levels (*p* < 0.001, Figure 2B) compared to the TNF-α/IFN-γ group. In contrast, only 20 μM of psilocybin resulted in significantly lower IL-6 (*p* < 0.05, Figure 2C) or IL-8 levels (*p* < 0.05, Figure 2D). No treatments significantly lowered MCP-1 levels; however, there appeared to be a trend where single treatment with 10 or 20 μM psilocybin appeared to lower MCP-1 levels compared to the TNF-α/IFN-γ group (*p* = N.S., Figure 2E). In contrast, 10 μM of psilocybin (*p* < 0.01), 20 μM of psilocybin (*p* < 0.0001), and the combination of 20 μM of psilocybin with 0.5 μM of curcumin (*p* < 0.01, Figure 2F) significantly lowered GM-CSF levels compared to the TNF-α/IFN-γ group. Out of all the treatments, the most effective treatment appeared to be 20 μM of psilocybin, which could significantly decrease TNF-α by −22.3×, IFN-γ by −33.7×, IL-6 by −2.5×, IL-8 by −1.5×, and GM-CSF by −6.2× compared to the TNF-α/IFN-γ group (Figure 2). Furthermore, psilocybin and curcumin co-treatment did not appear to have any synergistic anti-inflammatory effects.

**Figure 3:**
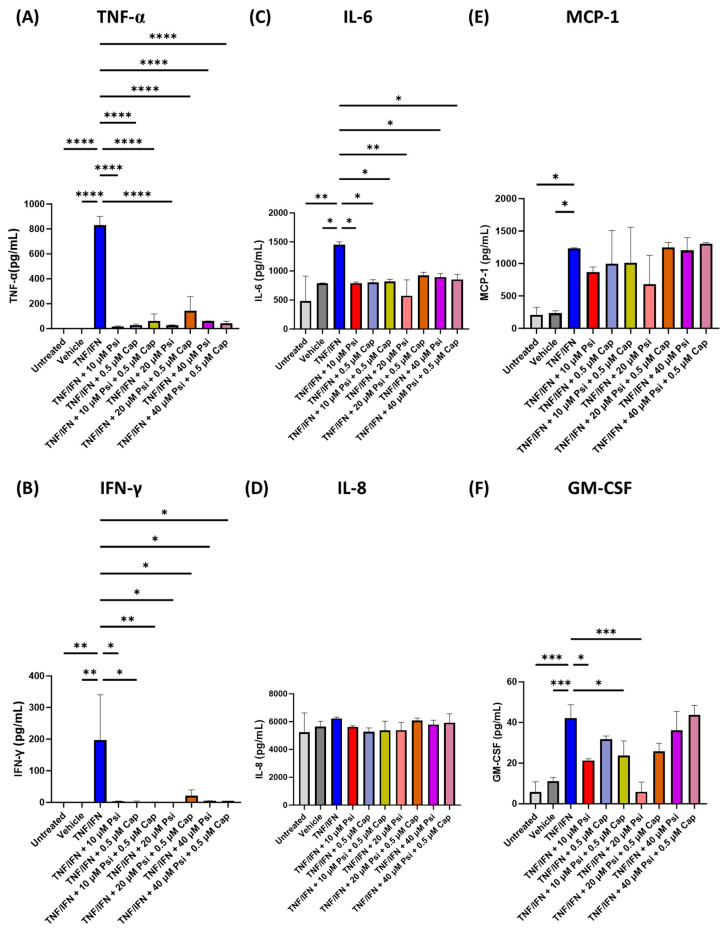
Psilocybin and capsaicin combinations alter protein levels of inflammatory cytokines, chemoattractants, and growth factors in human 3D EpiIntestinal tissue. Protein levels measured via ELISA for (**A**) TNF-α, (**B**) IFN-γ, (**C**) IL-6, (**D**) IL-8, (**E**) MCP-1, and (**F**) GM-CSF. Bars represent the mean ± SD. Data were analyzed with a one-way ANOVA test and Dunnett’s post hoc multiple comparison test compared to the TFN-α/IFN-γ group. Significance is indicated within the figures using the following scale: * *p* < 0.05, ** *p* < 0.01, *** *p* < 0.001, **** *p* < 0.0001. Cap, capsaicin; Psi, psilocybin.

Next, we tested the anti-inflammatory effects of psilocybin combined with capsaicin. TNF-α (*p* < 0.0001, Figure 3A), IFN-γ (*p* < 0.05, Figure 3B), IL-6 (*p* < 0.05, Figure 3C), MCP-1 (*p* < 0.05, Figure 3E), and GM-CSF (*p* < 0.001, Figure 3F) were significantly higher in the TNF-α/IFN-γ group compared to the untreated group or vehicle. However, IL-8 levels were not significantly altered in the TNF-α/IFN-γ group compared to the control (*p* = N.S., Figure 3D).

All single treatments of psilocybin or capsaicin and combination treatments resulted in significantly lower TNF-α (*p* < 0.0001, Figure 3A) IFN-γ (*p* < 0.05, Figure 3B), and IL-6 levels (*p* < 0.05, Figure 3C) compared to the TNF-α/IFN-γ group. However, no changes in any of the treatment groups were seen for either IL-8 (*p* = N.S., Figure 3D) or MCP-1 (*p* = N.S., Figure 3E). In contrast, 10 μM of psilocybin (*p* < 0.05), 10 μM of psilocybin combined with 0.5 μM of capsaicin (*p* < 0.05), and 20 μM of psilocybin (*p* < 0.001) significantly reduced GM-CSF levels compared to the TNF-α/IFN-γ group (Figure 3F). Once again, no synergistic effects were observed, and the strongest anti-inflammatory effects were seen when 20 μM of psilocybin was applied (Figure 3).

**Figure 4:**
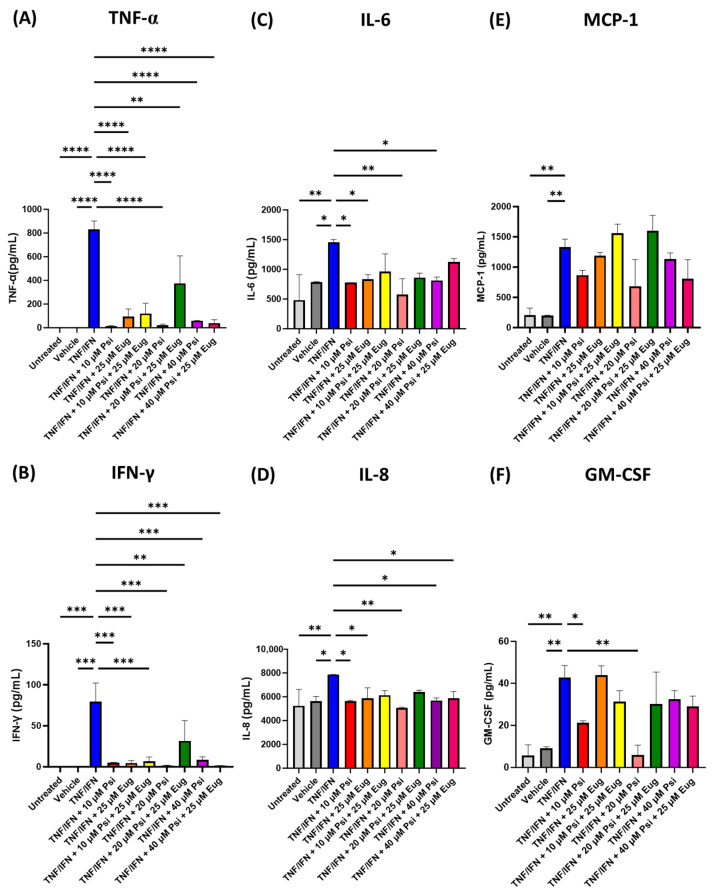
Psilocybin and eugenol combinations alter protein levels of inflammatory cytokines, chemoattractants, and growth factors in human 3D EpiIntestinal tissue. Protein levels measured via ELISA for (**A**) TNF-α, (**B**) IFN-γ, (**C**) IL-6, (**D**) IL-8, (**E**) MCP-1, and (**F**) GM-CSF. Bars represent the mean ± SD. Data were analyzed with a one-way ANOVA test and Dunnett’s post hoc multiple comparison test and compared to the TFN-α/IFN-γ group. Significance is indicated within the figures using the following scale: * *p* < 0.05, ** *p* < 0.01, *** *p* < 0.001, **** *p* < 0.0001. Eug, eugenol; Psi, psilocybin.

Next, we tested whether psilocybin and eugenol demonstrated synergistic anti-inflammatory effects on the inflammatory response seen in our 3D human EpiIntestinal inflammation model. Compared to the TNF-α/IFN-γ group, TNF-α (*p* < 0.0001, Figure 4A), IFN-γ (*p* < 0.001, Figure 4B), IL-6 (*p* < 0.05, Figure 4C), IL-8 (*p* < 0.05, Figure 4D), MCP-1 (*p* < 0.01, Figure 4E), and GM-CSF (*p* < 0.01, Figure 4F) were significantly higher in the TNF-α/IFN-γ group compared to the vehicle and untreated group.

While all treatments of psilocybin and/or eugenol significantly decreased TNF-α levels (*p* < 0.0001), the combination treatment of 20 μM of psilocybin and 25 μM of eugenol did not appear to be as effective in decreasing TNF-α levels compared to the TNF-α/IFN-γ group (*p* < 0.01, Figure 4A). Interestingly, single treatments of psilocybin resulted in the largest decrease in TNF-α levels (Figure 4A). Similar trends were seen in IFN-γ levels, whereby all treatments significantly reduced IFN-γ levels compared to the TNF-α/IFN-γ group (*p* < 0.001); however, the combination of psilocybin (20 μM) and eugenol (25 μM) did not appear to be as effective in lowering IFN-γ levels (*p* < 0.01, Figure 4B). Once again, the single treatments of psilocybin resulted in the largest decrease IFN-γ levels (Figure 4B). In contrast, IL-6 levels were only significantly lowered via treatment with 10 μM of psilocybin (*p* < 0.05), 25 μM of eugenol (*p* < 0.05), 20 μM of psilocybin (*p* < 0.01), and 40 μM of psilocybin (*p* < 0.01, Figure 4C) compared to the TNF-α/IFN-γ group. Similarly, IL-8 levels were only significantly lowered via treatment with 10 μM of psilocybin (*p* < 0.05), 25 μM of eugenol (*p* < 0.05), 20 μM of psilocybin (*p* < 0.01), 40 μM of psilocybin (*p* < 0.01, Figure 4C), as well as the combination of 40 μM of psilocybin and 25 μM of eugenol (*p* < 0.05, Figure 4D) compared to the TNF-α/IFN-γ group. Once again, none of the treatments significantly affected MCP-1 levels compared to the TNF-α/IFN-γ group (*p* = N.S., Figure 4E). Lastly, only 10 (*p* < 0.05) and 20 μM (*p* < 0.01) of psilocybin significantly decreased GM-CSF levels compared to the TNF-α/IFN-γ group (Figure 4F).

Once again, there was no obvious synergy between psilocybin and a TRP channel ligand, in this case, eugenol. Psilocybin alone demonstrated stronger or similar decreases in TNF-α and IFN-γ compared to psilocybin in combination with eugenol, except for 40 μM of psilocybin, which demonstrated larger decreases in the combination group. Similar to all of the previous TRP channel ligands tested, 20 μM of psilocybin appeared to have the most beneficial anti-inflammatory effects in the TNF-α/IFN-γ inflammatory response in 3D EpiIntestinal tissue with fold changes of −39.2× for TNF-α, −55.9× for IFN-γ, −2.5× for IL-6, −1.6× for IL-6, and −7.2× for GM-CSF (Figure 4).

### 3.3. 4-AcO-DMT and Eugenol Decrease Inflammation in Human 3D EpiIntestinal Tissue

**Figure 5:**
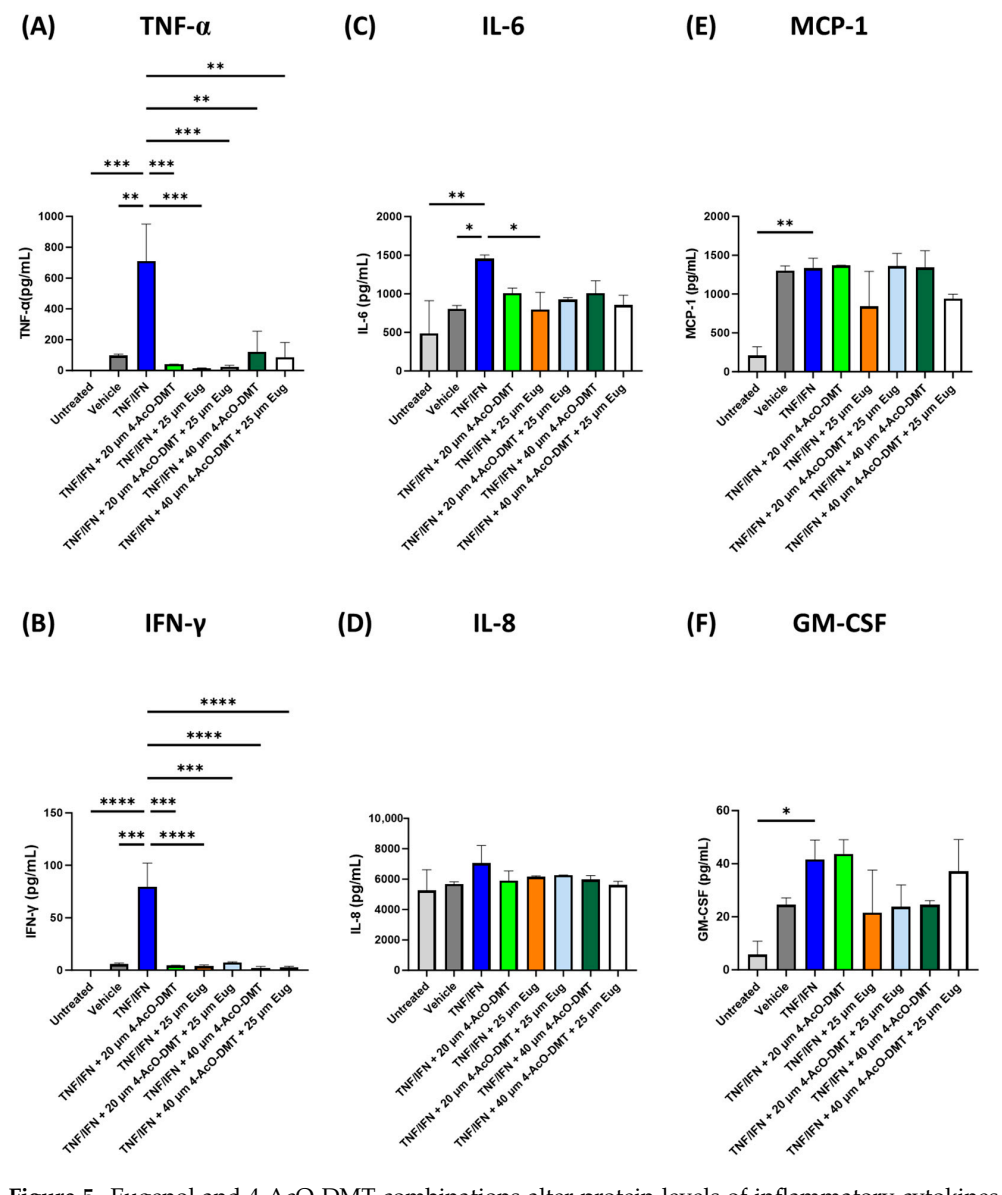
Eugenol and 4-AcO-DMT combinations alter protein levels of inflammatory cytokines, chemoattractants, and growth factors in human 3D EpiIntestinal tissue. Protein levels measured via ELISA for (**A**) TNF-α, (**B**) IFN-γ, (**C**) IL-6, (**D**) IL-8, (**E**) MCP-1, and (**F**) GM-CSF. Bars represent the mean ± SD. Data were analyzed with a one-way ANOVA test and Dunnett’s post hoc multiple comparison test compared to the TFN-α/IFN-γ group. Significance is indicated within the figures using the following scale: * *p* < 0.05, ** *p* < 0.01, *** *p* < 0.001, **** *p* < 0.0001. 4-AcO-DMT, 4-acetoxy-N,N-dimethyltryptamine; Eug, eugenol.

Due to 4-AcO-DMT’s ease of synthesis and low cost, we tested whether 4-AcO-DMT would have anti-inflammatory effects on the inflammatory response seen in our 3D human EpiIntestinal inflammation model, and whether 4-AcO-DMT had synergistic effects with eugenol. TNF-α/IFN-γ-exposed 3D EpiIntestinal tissue had significantly higher TNF-α (*p* < 0.01, Figure 5A), IFN-γ (*p* < 0.001, Figure 5B), and IL-6 levels (*p* < 0.05, Figure 5C) compared to the untreated group and vehicle, while IL-8 was not significantly different (*p* = N.S., Figure 5D); in addition, MCP-1 (*p* < 0.01, Figure 5E) and GM-CSF (*p* < 0.05, Figure 5F) was significantly upregulated compared to the untreated group but not the vehicle (*p* = N.S.)

While all treatments significantly reduced TNF-α (*p* < 0.01, Figure 5A) and IFN-γ (*p* < 0.001, Figure 5B) compared to the TNF-α/IFN-γ group, only eugenol (25 μM) significantly lowered IL-6 levels (*p* < 0.05, Figure 5C). In contrast, none of the treatments significantly lowered IL-8 (*p* = N.S., Figure 5D), MCP-1 (*p* = N.S., Figure 5E), or GM-CSF (*p* = N.S., Figure 5F) compared to the TNF-α/IFN-γ group.

Interestingly, 25 μM of eugenol appeared to have the strongest anti-inflammatory effects seen in TNF-α/IFN-γ-exposed 3D EpiIntestinal tissue. Eugenol resulted in fold changes of −49.7× for TNF-α, −20.8× for IFN-γ, and −1.8× for IL-6. While 4-AcO-DMT did reduce TNF-α and IFN-γ, none of the treatments significantly reduced IL-6, and cumulatively did not have as strong as an effect compared to eugenol. In addition, 4-AcO-DMT and eugenol did not show superior results to eugenol alone, and therefore did not show synergism (Figure 5).

### 3.4. Ketanserin and Eugenol Decrease Inflammation in Human 3D EpiIntestinal Tissue

**Figure 6:**
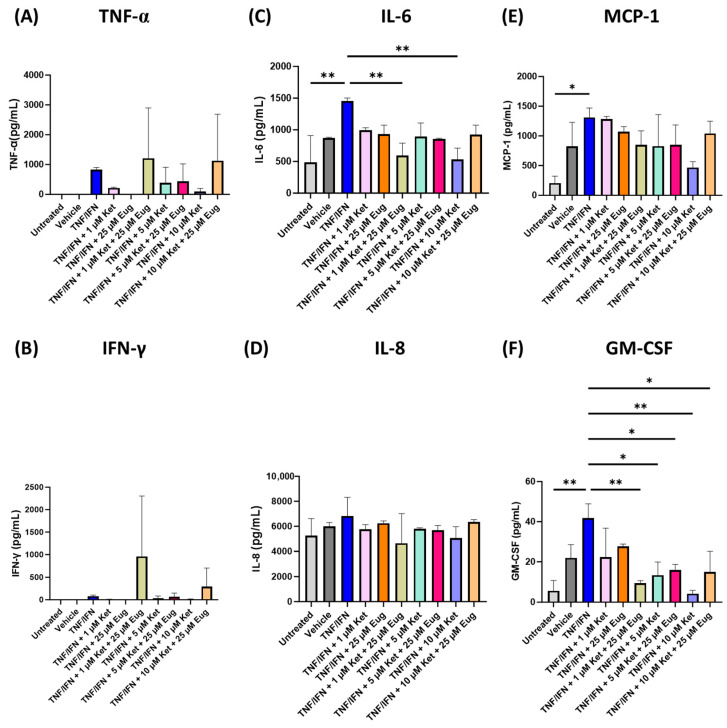
Ketanserin and eugenol combinations alter protein levels of inflammatory cytokines, chemoattractants, and growth factors in human 3D EpiIntestinal tissue. Protein levels measured via ELISA for (**A**) TNF-α, (**B**) IFN-γ, (**C**) IL-6, (**D**) IL-8, (**E**) MCP-1, and (**F**) GM-CSF. Bars represent the mean ± SD. Data were analyzed with a one-way ANOVA test and a Dunnett’s post hoc multiple comparison test compared to the TFN-α/IFN-γ group. Significance is indicated within the figures using the following scale: * *p* < 0.05, ** *p* < 0.01. Eug, eugenol; Ket, ketanserin.

Since ketanserin has shown anti-inflammatory effects in other models, we decided to test the effects of ketanserin alone and in combination with eugenol in our human 3D EpiIntestinal tissue TNF-α/IFN-γ model. Surprisingly, no significant differences were seen in either TNF-α (*p* = N.S., Figure 6A), or IFN-γ (*p* = N.S., Figure 6B). For IL-6, the TNF-α/IFN-γ group has significantly higher levels than the untreated group (*p* < 0.01), and while the TNF-α/IFN-γ appeared higher than the vehicle, it was not significantly higher (*p* = N.S., Figure 6C). Furthermore, 1 μM of ketanserin and 25 μM of eugenol (*p* < 0.01), as well as 10 μM of ketanserin (*p* < 0.01, Figure 6C) significantly lowered IL-6 levels compared to the TNF-α/IFN-γ group. In contrast, no significant changes were seen between any groups for IL-8 (*p* = N.S., Figure 6D). While MCP-1 levels were significantly higher in the TNF-α/IFN-γ group compared to the untreated group (*p* < 0.05) and appeared to be higher than the vehicle (*p* = N.S.), none of the treatments significantly lowered MCP-1 levels; however, 10 μM of ketanserin appeared to lower MCP-1 levels compared to the TNF-α/IFN-γ group (*p* = N.S., Figure 6E). Similarly, GM-CSF levels were significantly higher in the TNF-α/IFN-γ group compared to the untreated group (*p* < 0.01) and appeared to be higher than the vehicle (*p* = N.S.). Although neither 1 μM of ketanserin nor 25 μM of eugenol significantly lowered GM-CSF levels (*p* = N.S.), the combination of 1 μM of ketanserin and 25 μM of eugenol (*p* < 0.01), 5 μM of ketanserin (*p* < 0.01), 5 μM of ketanserin and 25 μM of eugenol (*p* < 0.05), 10 μM of ketanserin (*p* < 0.01), 10 μM of ketanserin, and 25 μM of eugenol (*p* < 0.05, Figure 6F) significantly lowered GM-CSF levels compared to the TNF-α/IFN-γ group.

Out of all the ketanserin treatments, only 1 μM of ketanserin and 25 μM of eugenol, as well as 10 μM of ketanserin were able to significantly lower both IL-6 and GM-CSF (*p* < 0.01, Figure 6). Between the two, the 10 μM of ketanserin dose had larger effects with fold changes of −2.7× for IL-6 and −9.9× for GM-CSF (Figure 6). Furthermore, 1 μM of ketanserin and 25 μM of eugenol increased TNF-α levels 1.5× fold and IFN-γ levels 12.6× fold; however, these changes were not significant (*p* = N.S., Figure 6). Eugenol had the largest fold change of −37.3× for TNF-α and −55.6× for IFN-γ, but was not significant (*p* = N.S., Figure 6). Together, this would suggest that either eugenol or ketanserin alone would provide the best anti-inflammatory effects.

## 4. Discussion

In this study, we aimed to investigate the anti-inflammatory effects of select 5-HT2A and TRP channel ligands on the inflammatory response in human 3D EpiIntestinal tissue. To induce an inflammatory response, we used MatTek’s 3D intestinal tissue model, which has been extensively used to study inflammation as well as other diseases [33], and exposed the 3D EpiIntestinal tissue to 10 ng/mL of TNF-α/IFN-γ for different amounts of time to measure COX-2 induction, which has been previously shown to recapitulate an inflammatory response in 3D tissue [34,35].

COX-2, which is the inducible form of cyclooxygenase, was utilized to measure the inflammatory response as COX-2 is the key initiator of the inflammatory response in peripheral tissues by converting arachidonic acid into proinflammatory prostaglandins to regulate homeostatic functions, mediate pathogenic mechanisms, and importantly, to induce the production of other proinflammatory compounds [36,37]. After 12 h of 10 ng/mL of TNF-α/IFN-γ exposure, we were able to strongly upregulate the expression of COX-2 (Figure 1), and therefore utilized this concentration and timepoint to test the efficacy of 5-HT2A and TRP channel ligands as anti-inflammatory therapeutics.

Initially, we tested the efficacy of psilocybin combined with curcumin (Figure 2). While both TNF-α and IFN-γ were significantly higher, as measured by ELISA, all treatments significantly lowered TNF-α and IFN-γ levels (Figure 2A,B). No synergistic effects were seen between psilocybin and curcumin to reduce either TNF-α and IFN-γ levels; however, all treatments were able to significantly reduce levels of both cytokines (Table 1).

In previous studies, TNF-α overexpression in mice had been shown to result in the development of IBD pathologies like Crohn’s disease (CD), giving evidence for TNF-α as one of the causative factors in IBD pathogenesis [38]. Furthermore, TNF-α plays a prominent role in IBD and intestinal inflammation by regulating several cellular functions, including the synthesis of inflammatory mediators, cell proliferation, survival, and cell death. The largest decrease in fold change for TNF-α was when 0.5 μM of curcumin was exposed to the 3D tissue (Table 1). By decreasing TNF-α levels, IBD can be ameliorated.

IFN-γ has been suggested as a possible mediator in CD in a synergistic combination with TNF-α [39]. IFN-γ activates JAK1 and JAK2, which in turn activates STAT1 and consequent cellular responses [40]. Additionally, IFN-γ may cause cytotoxicity in intestinal epithelial cells (IECs), including apoptosis and necroptosis [41,42,43]. High IFN-γ and raised STAT1 activity is usually caused by CD lesions [44,45,46]. The synergistic effect of IFN-γ and TNF-α contributes to IEC death and epithelial barrier breakdown [47,48]. STAT1 emerges as a crucial mediator driving IEC death produced by the additive actions of IFN-γ and TNF-α. Furthermore, JAK1 and JAK2 have been identified as the primary drivers of IEC death induced by the combined action of IFN-γ and TNF-α [35]. In our first experiment, 20 μM of psilocybin provided the largest decrease in IFN-γ levels with a −25.8× fold change (Table 1), suggesting that psilocybin alone can better reduce and prevent the downstream signaling of IFN-γ.

Next, we looked at IL-6 levels (Figure 2D). IL-6 is a pro-inflammatory cytokine that forms a soluble complex with its receptor to induce the synthesis of acute phase reactants and promotes the development of inflammatory processes [49]. Studies have found that in animal models of chronic intestinal inflammation, inhibiting the IL-6 trans-signaling pathway has shown therapeutic promise [50]. While TNF-α/IFN-γ treatment significantly increased IL-6 levels, only 20 μM of psilocybin reduced IL-6 levels. Curcumin appeared to have no effect on IL-6 levels. While some in vitro studies have shown that curcumin is able to reduce IL-6 levels [51], a recent meta-analysis has shown that the oral consumption of curcumin does not affect the circulating levels of IL-6 [52].

Similarly, IL-8 levels were shown to be increased in the TNF-α/IFN-γ treatment group and only 20 μM of psilocybin ameliorated IL-8 levels (Figure 2D). Particularly, neutrophils produce a large quantity of IL-8 in the presence of ulcerated and inflammatory mucosa. In situ hybridization and immunohistochemistry studies demonstrated that macrophages are the key producers of IL-8 in ulcerated tissue associated with IBD. Additionally, the CD14 marker expression is directly linked to macrophage IL-8 production in IBD. Usually, CD14 is not present in intestinal macrophages; however, it appears in a higher number of cells in IBD, signaling that monocytes have recently been imported from the circulation [35]. Similarly, IL-8 is not decreased in humans after the consumption of curcumin [52]. Surprisingly, circulating IL-8 levels are actually higher after curcumin exposure [52].

While MCP-1 levels were significantly increased in the TNF-α/IFN-γ treatment group compared to the controls, none of the psilocybin and/or curcumin treatments significantly lowered MCP-1 levels compared to TNF-α/IFN-γ group (Figure 2E). However, both single doses appeared to lower MCP-1 levels by −1.5× or −1.9× fold for 10 and 20 μM of psilocybin, respectively (*p* = N.S., Table 1). MCP-1 functions to attract monocytes and macrophages to inflamed tissue and is generated by both intestinal epithelial cells and resident macrophages. As a result, MCP-1 alters the makeup of resident macrophages and may impact dendritic cell and T cell development [53]. While psilocybin does appear to reduce MCP-1 levels and thus prevent macrophage recruitment, curcumin does not appear to have any effects, and may even blunt the effect shown by psilocybin (*p* = N.S., Figure 2E). This is a surprising finding as curcumin is known to inhibit MCP-1 production by blocking the protein kinase C-mediated activation of ERK and NF-κB signaling in many monocytes and macrophages cell lines [54,55]. However, our 3D tissue model does not include any immune cells, and potentially, these effects are not seen on epithelial intestinal cells.

Lastly, we measured GM-CSF levels in response to various levels of psilocybin and/or curcumin after inflammatory-induction with TNF-α/IFN-γ. GM-CSF has been discovered as a crucial regulator of intestinal macrophage activation in individuals with IBD and animals with DSS-induced colitis [56]. GM-CSF promotes the development and orientation of inflammatory intestinal macrophages and can also decrease wound repair transcriptional programs. Notably, during intestinal inflammation, GM-CSF is largely generated by group 3 inborn lymphoid cells (ILC3s), and a substantial positive connection has been established between ILC or CSF2 transcripts and M1 macrophage profiles in IBD mucosal biopsies [57]. Interestingly, psilocybin significantly lowered GM-CSF levels compared to the TNF-α/IFN-γ group at both 10 and 20 μM doses by −1.6× (*p* < 0.01) and −6.2× fold (*p* <0.0001, Table 1), respectively. To the best of our knowledge, no other studies have shown that psilocybin can alter GM-CSF levels; however, the stimulation of serotonin signaling during inflammation has been shown to inhibit GM-CSF in a P38- and PI3K-dependent pathway [58]. Presumably, psilocybin would act through a similar mechanism.

In our next experiment, we tested psilocybin and capsaicin, both as single treatments and as co-treatment for synergistic effects. Capsaicin alone significantly reduced TNF-α levels by −28.8×, IFN-γ by −74.9×, and IL-6 levels by −1.8× fold, while IL-8, MCP-1, and GM-CSF levels were not affected (Table 1). This is in line with our previous study in human small intestinal epithelial cells (HSIEC), which showed that capsaicin is able to inhibit TNF-α production [12] and previous studies showing capsaicin reduced TLR/NFκB-mediated TNF-α production [59]. In addition, we demonstrated here that capsaicin can reduce IFN-γ, and IL-6 levels, which has not been shown before. This builds on previous studies that have shown the anti-inflammatory effects of capsaicin in human colon cancer cell lines [60] and porcine epithelial cell lines [59]. Interestingly, we did not see any major effects of IL-8 levels, which was inhibited, albeit minimally, in previous studies utilizing an LPS-induced inflammatory response in porcine epithelial cells [59]. While capsaicin has been shown to inhibit MCP-1 levels in macrophages through TRPV1 activation [61,62], these effects are not seen here in our study or other studies investigating the inflammatory responses of epithelial cells [63].

Compared to psilocybin, capsaicin appeared to be either equally effective or less effective in reducing IFN-γ, IL-6, TNF-α, and GM-CSF levels (Table 1). Furthermore, when paired together, their effects appeared blunted or equal to treatment with psilocybin at the same dose. Due to these findings, psilocybin appears to be a more effective treatment for IBD than capsaicin.

Next, we tested the efficacy of psilocybin and eugenol to prevent the inflammatory response induced by TNF-α/IFN-γ. While our previous studies have demonstrated psilocybin and eugenol have synergistic effects on COX-2 and IL-6 within HSIEC in vitro [12], as well as synergistic effects on reducing COX-2 and IL-6 levels within the LPS-induced murine brain inflammation [22], in this study, we only saw synergistic effects at the highest dose of psilocybin (40 μM) combined with eugenol (25 μM) (Table 1). With the combined treatment of psilocybin (40 μM) and eugenol (25 μM), there was a fold change of −21.0× and −62.5× for TNF-α and IFN-γ, respectively, which was superior to both psilocybin’s induced fold changes of −13.7× and −9.5×, or eugenol’s induced fold changes of −8.8× and −17.0× for TNF-α and IFN-γ, respectively (Table 1). While these data may initially be promising, it is important to note that lower doses of psilocybin appear to have more beneficial results. When treated with 10 or 20 μM of psilocybin, there was a much larger decrease in TNF-α levels, while IL-6 and GM-CSF levels were also decreased, which was not seen in the combination of psilocybin and eugenol (Table 1). Once again, the dose that appeared to have the strongest anti-inflammatory effects in our model was psilocybin at a dose of 20 μM (Table 1).

In both our previous in vitro and in vivo studies testing psilocybin and eugenol [61,62], we noted synergistic effects in IL-6, but no signs of synergistic effects were seen here. Psilocybin acts primarily through the 5-HT2A receptor, likely acting through Gα_q/11_, Gα_i/o_, and/or β-arrestin-2 to inhibit NF-κB signaling [64,65,66]. In contrast, eugenol can interact with TRPV1 to induce CaMKK2 signaling [67], which suppresses chemokine production in multiple myeloid subsets [68]. Due to the different mechanisms of action and previous studies, we believed there would be synergistic effects between eugenol and psilocybin, making the results shown here surprising. However, the lack of any synergistic effects could be due to the model used. Importantly, 3D in vitro models are known to be more resistant to pharmaceutical treatments then monolayer cells, and the higher doses required to see synergistic effects on TNF-α and IFN-γ could be due to this phenomenon [69]. Potentially, at higher doses, a synergistic effect would be seen between psilocybin and eugenol on IL-6 levels. It is difficult to predict which model would more accurately represent what would occur in vivo in humans.

Since psilocybin did not appear to have any synergistic effects with either curcumin, capsaicin, or eugenol, we decided to test whether 4-AcO-DMT could synergistically inhibit the inflammatory response in our 3D EpiIntestinal tissue model. The 5-HT2A receptor exhibits biased agonism that can result in ligand-specific effects due to different receptor binding pockets [70] and downstream signaling [71]. As such, it is possible that ligands other than psilocybin can produce synergistic effects with eugenol. We chose not to test capsaicin or curcumin due to their limited ability in altering IL-6, IL-8, MCP-1, or GM-CSF levels (Table 1). Furthermore, 4-AcO-DMT is an acetylated form of psilocin and can be inexpensively synthesized; therefore, if 4-AcO-DMT presents similar anti-inflammatory results, 4-AcO-DMT could be used instead of psilocybin.

Our results show 4-AcO-DMT only significantly affects TNF-α and IFN-γ levels; however, it did not significantly affect IL-6, IL-8, MCP-1, or GM-CSF (Table 1). As such, psilocybin would likely be a better potential therapeutic than 4-AcO-DMT. Similar results were seen in our previous monolayer cell model [12]. Due to limited studies on 4-AcO-DMT, the difference in signaling induced by psilocybin and 4-AcO-DMT is not known; however, the difference is likely due to the biased signaling which is a result of the extended binding pocket that has a strong affinity for psilocybin [11,70]. Furthermore, no synergistic effects were seen between 4-AcO-DMT and eugenol.

Next, we tested potential synergies between ketanserin and eugenol. Ketanserin is a selective antagonist of the 5-HT2A and has been shown to inhibit M2 macrophage polarization, migration, and NF-κB activation, resulting in ameliorated intestinal mucosa architecture [14]. Ketanserin’s anti-inflammatory effects are known to be mediated through MEK/ERK signaling to reduce nitrosative stress and inhibit IL-6 production [72], likely through Stat3 signaling [73]. In our study, IL-6 levels were significantly reduced by ketanserin at 10 μM (Table 1). Furthermore, 1 μM of ketanserin combined with 25 μM of eugenol has a fold change of −2.5× and −4.4× for IL-6 and GM-CSF, respectively (Table 1). As neither treatment with eugenol (25 μM) nor ketanserin (1 μM) alone resulted in significant decreases in either IL-6 or GM-CSF, this could suggest ketanserin and eugenol synergistically pair to reduce IL-6 and GM-CSF levels. In contrast, none of the treatments resulted in a significant decrease in TNF-α, IFN-γ, IL-8, or MCP-1 levels (Table 1). While ketanserin is known to reduce pro-inflammatory cytokines by inhibiting 5-HT2A signaling, these affects are primarily mediated through monocytes and macrophages [72,74,75], and are therefore in alignment with our data.

In contrast, little is known about ketanserin’s effect on GM-CSF. One study has shown serotonin signaling has induced GM-CSF, which was inhibited by ketanserin in megakaryocyte cells [76]. In contrast, the selective serotonin receptor uptake inhibitor, fluoxetine, has shown pleiotropic effects on GM-CSF. Under physiological conditions, fluoxetine-induced GM-CSF production in macrophages, whereas when stimulated with LPS, fluoxetine, which increases serotonin signaling, completely stopped GM-CSF production through 5-HT2B activation [58,77]. This is contradictory to our findings; however, our model does not include macrophages. Potentially, these differences in response to serotonin signaling are due to different cell types being tested.

Previous studies have shown psychedelic mushroom extracts have similar anti-inflammatory effects. Ethanol extracts of *Psilocybe natalensis* were shown to reduce LPS-induced nitric oxide production and increase cell viability in RAW 264.7 murine macrophages [7]. Furthermore, both water and ethanol extracts reduced prostaglandin, TNF-α, and IL-1β production [7]. In contrast, only the water extracts reduced IL-10 production [7]. In addition, four psychedelic mushroom water extracts, including *Panaeolus cyanescens*, *Psilocybe natalensis*, *Psilocybe cubensis*, and *Psilocybe cubensis* leucistic A+ strain, were tested on human U937 macrophage cells [8]. While all strains reduced TNF-α and IL-1β levels, only the *P. cubensis* A+ strain reduced COX-2 levels, and only *P. natalensis* and *P. cubensis* reduced IL-6 levels in LPS-induced human macrophages [8]. While our study did not utilize psychedelic mushroom extracts, we similarly saw large decreases in multiple inflammatory cytokines, growth factors, and chemokines (Table 1). Furthermore, our data adds that these effects are not just on macrophages but also show the ability to reduce the inflammatory response seen in epithelial intestinal cells, while also showing the active ingredient in psychedelic mushroom extracts, psilocybin, can lower MCP-1 and GM-CSF production. The effects of psychedelic mushrooms may be compounding in IBD as the inflammatory response in epithelial cells are inhibited, the production of chemoattractants is reduced, and any recruited macrophages would be inhibited as well. Together, this suggests psilocybin or psychedelic mushroom extracts could play a significant role in IBD therapeutics. Further research should determine whether the use of psychedelic mushroom extracts or psilocybin is more efficacious.

While psilocybin mushroom extracts and psilocybin provide potent anti-inflammatory effects, it is important to note that the exact mechanism has not been determined. Although the anti-inflammatory effects of psychedelic mushroom extracts are attributed to psilocybin, there are likely other compounds in psychedelic mushrooms that affect inflammatory responses. Furthermore, the exact mechanism of how psilocybin inhibits the inflammatory response in epithelial cells is currently unknown. The effects of psilocybin are likely mediated through 5-HT2A, but could also be influenced by other receptors known to be affected by psilocybin, including 5-HT2B, 5-HT2C, or 5-HT1A [78]. The most plausible signaling of psilocybin involves the biased activation of the 5-HT2A receptor resulting in β-arrestin2-dependent signaling, which negatively regulates NF-κB [65,66]. However, recent evidence indicates that some effects may be mediated through glucocorticoid signaling pathways [79].

It is important to note that there has been an increasing number of studies exploring the potential of serotonin receptor ligands as anti-inflammatory agents. This is partly due to approximately 95% of all serotonin produced within the body is synthesized by enterochromaffin cells, which are found in the intestines and regulate gut microbiota [80]. Within the gut, serotonin is known to modulate gut motility, secretion, metabolic homeostasis, and intestinal permeability, while the role in inflammation and inflammatory diseases is still developing [80,81]. Specifically, the regulation of the intestinal barrier and gut microbiota can be tied to the serotonergic activation of immune cells [82], but only 5-HT1, 5-HT3, and 5-HT4 receptors have been thoroughly studied [83]. The few studies that have examined the 5-HT2A receptor has been limited to specific ligands, including (R)-DOI, which has different binding than psilocybin to 5-HT2A [11,70]. Nevertheless, (R)-DOI could significantly reduce TNF-induced *mcp-1*, *il-6*, and *il-1beta* transcripts [84]. In addition, knockout of the *htr2b* gene which encodes the 5-HT2B receptor demonstrated 5-HT2B prevents the development of colitis, suggesting the activation of the 5-HT2B receptor could help prevent IBD [85]. Due to these promising findings, further research should determine the role of the 5-HT2 receptors in regulating inflammation and IBD with special emphasis on the effects of psilocybin.

While the current study displays the effects of a wider range of pro-inflammatory cytokines, chemokines, and growth factors, there are still limitations to this study. Previous research has focused on cellular models [9,12], and while this study utilized 3D tissue, in vivo animal models are required to test and analyze inflammation, which is a systemic process. The current study does not take into account how various other immune cells will act on the intestinal epithelium and modulate the effects of psilocybin and other compounds. Furthermore, this model creates an inflammatory response utilizing TNF-α/IFN-γ; however, numerous cytokines and interconnecting signaling pathways between intestinal epithelial cells and immune cells determine the effects of psilocybin and other potential therapeutics on inflammation and IBD [86]. In addition, due to the marked biased agonism at the 5-HT2A receptor, testing other ligands and various timepoints should be carried out as the effects cannot be generalized [12,70].

There are legitimate health concerns regarding using psychedelics as therapeutics due to the hallucinogenic effects of psychedelics and the unknown long-term effects. Importantly, the acute and even long-term effects of infrequent psilocybin use have been shown to be minimal when the set and setting is controlled [87,88]. When discussing the use of psilocybin for IBD, non-hallucinogenic doses can be used; however, frequent doses would likely be utilized as the effects are acute. The frequent use of sub-hallucinogenic doses has become increasingly popular, which is known as microdosing; however, the long-term effects are not known. Furthermore, while adverse health effects are rarely reported, multiple authorities recognize the need to study the safety of microdosing as this would result in the consistent modulation of serotonergic signaling [89,90,91]. While both microdosing and selective serotonin reuptake inhibitors (SSRI) effect the 5-HT signaling pathways, their actions are markedly different, and therefore, making generalizations about the lack of safety contraindications and side effects of SSRI’s compared to microdosing is currently unfounded [92]; however, future studies are required to understand the exact effects and potential long term consequences.

Concerns regarding the safety and tolerability of psychedelics have been the subject of recent reviews [93,94]. Despite common opinion, psilocybin has not been shown to be neurotoxic with either enteral or parenteral administration [95]. Furthermore, the clinical dose of 25 mg is well below the calculated LD_50_, which is over 2 g/kg [95]. In contrast, there is reason to believe psilocybin can be harmful to the cardiovascular system. Both excess serotonin (e.g., carcinoid tumors) and prolonged exposure to drugs with high agonist functional activity at the 5-HT2B receptor are known to increase myofibroblast mitogenesis and glycosaminoglycan deposition within heart valves. This profibrotic process interferes with normal valvular function, leading to a thickening of cardiac valve leaflets, subvalvular apparatus, and the impaired motion of one or more valves, as diagnosed by echocardiography [96]. This has included FDA-approved drugs like ergotamine for migraine headaches, pergolide for Parkinson’s disease, and cabergoline for hyperprolactinemia due to off-target 5-HT2B agonist activity [97]. Due to the agonism of 5-HT2B receptors in the cardiac valvulae being correlated with valvular heart disease (VHD), the FDA has issued a First Draft Guidance on Clinical Trials with Psychedelic Drugs emphasizing the importance of evaluating the association of VHD with psychedelic drug exposure [97].

Importantly, psilocin has been shown to bind to multiple receptors, including 5-HT2C, 5-HT1A, and monoamine transporters [98]; however, the binding properties of psilocin to 5-HT2B is currently unknown. Due to the binding of similar tryptamine analogues, psilocin is predicted to be a weak agonist of the 5-HT2B receptor [99]. While the EC**_50_** for psilocin binding to 5-HT2B has not been calculated, it is known to be higher than 20 μM [98]. As the oral clinical dose (25 mg) has been shown to have a mean maximal psilocin blood concentration of 3.82 nM [100], it is unlikely that physiological levels of psilocin induce much of an effect on 5-HT2B, if any. However, it is still imperative for future psychedelic treatments to rule out any association between psilocybin use and VHD in clinical trials prior to adoption as a therapeutic.

Furthermore, the use of psilocybin for IBD also presents potential limitations and side effects. While psilocybin shows promise for IBD, as it has been shown to be a potent anti-inflammatory at sub-hallucinogenic doses [9], there are potential side effects within the intestines. Serotonin is an important signaling molecule and is known to be involved in intrinsic reflexes, epithelial secretion, and vasodilation within the gut [101]. Specifically, 5-HT2A is found in smooth muscle cells, neurons, enterocytes, and Paneth cells. Moreover, 5-HT2A agonism supports the motility and maintenance of cells populations and enteric functions [102]. Due to serotonin signaling modulating intestinal motility and secretion, utilizing psilocybin as an anti-inflammatory in IBD could result in gastrointestinal discomfort. Furthermore, serotonin signaling in the gut is not fully known, and therefore, a wide range of unknown side effects could occur. Clinical trials should test the efficacy and possible side effects of psilocybin in IBD.

While studying the effects of ketanserin is interesting from a scientific perspective to better understand 5-HT2A signaling, the use of ketanserin as an IBD therapeutic is unlikely. Ketanserin is used to manage preeclampsia, treat hypertension, and chronic ulcers; however, now there are known adverse effects of ketanserin. This includes prolonging the cardiac QT interval, which results in cardiac arrythmias [15]; causing fatigue; headaches; insomnia; and dyspepsia [16], as well as orthostatic hypotension [17]. Due to these adverse effects, the clinical use of ketanserin as an anti-inflammatory for IBD is likely contraindicated.

In addition, capsaicin has shown great promise in cellular models of inflammation; however, *in vivo*, capsaicin appears to worsen IBD. A recent study has shown that orally consumed capsaicin reduced Firmicutes and increased Bacteroides, which caused intestinal permeability and endotoxemia. Due to the effects of capsaicin being modulated by the gut’s microbiota, oral in vivo use of capsaicin exacerbates inflammation, instead of improving conditions [103]. Furthermore, even low doses of capsaicin can result in cell cytotoxicity [12], while unpleasant side effects at doses as low as 10 mg can induce intestinal cramping and discomfort [31].

In contrast, TRP ligands curcumin and eugenol do not have adverse reactions that would limit their use. Curcumin’s acceptable daily intake is up to 3 mg/kg of body weight [26], while eugenol has been declared by WHO to be generally recognized as safe and non-mutagenic. Unfortunately, no synergistic effects were seen with serotonergic receptors ligands within this study; however, there may be synergistic effects in vivo as curcumin and eugenol are known to act on macrophages [23,104].

## 5. Conclusions

This study has systematically tested the effects of serotonin receptor ligands, psilocybin, 4-AcO-DMT, and ketanserin, paired with TRP channel ligands, capsaicin, curcumin, and eugenol on the TNF-α/IFN-γ-induced inflammatory response within 3D EpiIntestinal tissue. While minimal synergistic effects were seen between 5-HT2A and TRP channel ligands, this study has provided evidence that psilocybin and eugenol can lower TNF-α, IFN-γ, IL-6, IL-8, MCP-1, and GM-CSF in a human 3D EpiIntestinal model. Future studies should further test these compounds as they may provide potent anti-inflammatory effects in human inflammatory diseases, including inflammatory bowel disease.

## Figures and Tables

**Table 1:** Summary of fold changes of TNF-α, IFN-γ, IL-6, IL-8, MCP-1, and GM-CSF for each treatment of psilocybin, 4-AcO-DMT, ketanserin, capsaicin, curcumin, and eugenol compared to the TNF-α/IFN-γ-exposed 3D EpiIntestinal tissue.

Treatment	TNF-α	IFN-γ	IL-6	IL-8	MCP-1	GM-CSF
TNF/IFN + 10 μM Psi	−55.0× ****	−16.7× *	−1.9× *	−1.4× *	−1.5×	−2.0× *
TNF/IFN + 25 μM Eug	−8.8× ****	−17.0× *	−1.7× *	−1.3× *	−1.1×	1.0×
TNF/IFN + 10 μM Psi + 25 μM Eug	−6.9× ****	−11.5× **	−1.5×	−1.3×	1.2×	−1.4×
TNF/IFN + 0.5 μM Cur	−48.2× ***	−23.2× ***	−1.4×	−1.2×	−1.0×	−1.1×
TNF/IFN + 10 μM Psi + 0.5 μM Cur	−30.7× ***	−21.7× ***	−1.3×	−1.2×	1.1×	1.2×
TNF/IFN + 0.5 μM Cap	−28.8× ****	−74.9× *	−1.8× *	−1.2×	−1.2×	−1.3×
TNF/IFN + 10 μM Psi + 0.5 μM Cap	−13.2× ****	−196.9× **	−1.8× *	−1.2×	1.2×	−1.8× *
TNF/IFN + 20 μM Psi	−39.2× ****	−55.9× *	−2.5× **	−1.6× **	−2.0×	−7.2× ***
TNF/IFN + 20 μM Psi + 25 μM Eug	−2.2× ****	−2.5× *	−1.7×	−1.2×	1.2×	−1.4×
TNF/IFN + 20 μM Psi + 0.5 μM Cur	−3.8× ***	−13.6× ***	−1.7×	−1.3×	−1.4×	−2.0× **
TNF/IFN + 20 μM Psi + 0.5 μM Cap	−5.8× ****	−9.0× *	−1.6× *	−1.0×	−1.0×	−1.6×
TNF/IFN + 40 μM Psi	−13.7× ****	−9.5× *	−1.8× *	−1.4× *	−1.2×	−1.3×
TNF/IFN + 40 μM Psi + 25 μM Eug	−21.0× ****	−62.5× *	−1.3×	−1.3× *	−1.6×	−1.5×
TNF/IFN + 40 μM Psi + 0.5 μM Cap	−20.0× ****	−36.7× *	−1.7× *	−1.1×	1.1×	1.0×
TNF/IFN + 20 μm 4-AcO-DMT	−17.5× ***	−17.9× ***	−1.4×	−1.2×	−1.0×	1.1×
TNF/IFN + 25 μM Eug	−49.7× ***	−20.8× ****	−1.8× *	−1.1×	−1.6×	−1.9×
TNF/IFN + 20 μm 4-AcO-DMT + 25 μm Eug	−30.0× ***	−10.6× ***	−1.6×	−1.1×	1.0×	−1.8×
TNF/IFN + 40 μm 4-AcO-DMT	−5.9× **	−36.0× ****	−1.4×	−1.2×	1.0×	−1.7×
TNF/IFN + 40 μm 4-AcO-DMT + 25 μm Eug	−8.8× **	−30.6× ****	−1.7×	−1.3×	−1.4×	−1.1×
TNF/IFN + 1 μM Ket	−3.6×	−9.7×	−1.5×	−1.2×	−1.0×	−1.9×
TNF/IFN + 25 μM Eug	−37.3×	−55.6×	−1.6×	−1.1×	−1.2×	−1.5×
TNF/IFN + 1 μM Ket + 25 µM Eug	1.5×	12.6×	−2.5× **	−1.5×	1.5×	−4.4× **
TNF/IFN + 5 μM Ket	−2.1×	−2.2×	−1.6×	−1.2×	−1.6×	−3.1× *
TNF/IFN + 5 μM Ket + 25 µM Eug	−1.9×	−1.2×	−1.7×	−1.2×	−1.5×	−2.6× *
TNF/IFN + 10 μM Ket	−8.8×	−7.8×	−2.7× **	−1.3×	−2.8×	−9.9× **
TNF/IFN + 10 μM Ket + 25 µM Eug	1.4×	3.7×	−1.6×	−1.1×	−1.3×	−2.8× *

Note: Statistical analysis corresponds to significance in Figure 2, Figure 3, Figure 4, Figure 5 and Figure 6: one-way ANOVA followed by Dunnett’s post hoc test compared to TNF-α/IFN-γ group. * *p* < 0.05, ** *p* < 0.01, *** *p* < 0.001, **** *p* < 0.0001. 4-AcO-DMT, 4-acetoxy-N,N-dimethyltryptamine; Cap, capsaicin; Cur, curcumin; Eug, eugenol; Ket, ketanserin; Psi, psilocybin.

## Data Availability

Data is contained within the article and supplementary material.

## Supplementary Materials

The following supporting information can be downloaded at: https://www.mdpi.com/article/10.3390/life13122345/s1, Figure S1. Original Western blots of proteins from human 3D EpiIntestinal tissue showing COX-2 at molecular weight of 72 kDa. Red arrow indicates bands shown in Figure 1B. Figure S2. Original Western blots of proteins from human 3D EpiIntestinal tissue showing GAPDH at molecular weight of 36 kDa. Red arrow indicates bands shown in Figure 1B.

## References

[1] Lee J.Y., Wasinger V.C., Yau Y.Y., Chuang E., Yajnik V., Leong R.W. (2018). Molecular Pathophysiology of Epithelial Barrier Dysfunction in Inflammatory Bowel Diseases. Proteomes.

[2] Laukoetter M.G., Nava P., Nusrat A. (2008). Role of the intestinal barrier in inflammatory bowel disease. World J. Gastroenterol..

[3] Abdulla M., Mohammed N. (2022). A Review on Inflammatory Bowel Diseases: Recent Molecular Pathophysiology Advances. Biol. Targets Ther..

[4] Cai Z., Wang S., Li J. (2021). Treatment of Inflammatory Bowel Disease: A Comprehensive Review. Front. Med..

[5] Alatab S., Sepanlou S.G., Ikuta K., Vahedi H., Bisignano C., Safiri S., Sadeghi A., Nixon M.R., Abdoli A., Abolhassani H. (2020). The global, regional, and national burden of inflammatory bowel disease in 195 countries and territories, 1990–2017: A systematic analysis for the Global Burden of Disease Study 2017. Lancet Gastroenterol. Hepatol..

[6] Mak W.Y., Zhao M., Ng S.C., Burisch J. (2020). The epidemiology of inflammatory bowel disease: East meets west. J. Gastroenterol. Hepatol..

[7] Nkadimeng S.M., Nabatanzi A., Steinmann C.M.L., Eloff J.N. (2020). Phytochemical, Cytotoxicity, Antioxidant and Anti-Inflammatory Effects of Psilocybe Natalensis Magic Mushroom. Plants.

[8] Nkadimeng S.M., Steinmann C.M.L., Eloff J.N. (2021). Anti-Inflammatory Effects of Four Psilocybin-Containing Magic Mushroom Water Extracts in vitro on 15-Lipoxygenase Activity and on Lipopolysaccharide-Induced Cyclooxygenase-2 and Inflammatory Cytokines in Human U937 Macrophage Cells. J. Inflamm. Res..

[9] Flanagan T.W., Nichols C.D. (2018). Psychedelics as anti-inflammatory agents. Int. Rev. Psychiatry Abingdon Engl..

[10] Kalkman H.O. (2023). Inhibition of Microglial GSK3β Activity Is Common to Different Kinds of Antidepressants: A Proposal for an In Vitro Screen to Detect Novel Antidepressant Principles. Biomedicines.

[11] Klein A.K., Chatha M., Laskowski L.J., Anderson E.I., Brandt S.D., Chapman S.J., McCorvy J.D., Halberstadt A.L. (2021). Investigation of the Structure-Activity Relationships of Psilocybin Analogues. ACS Pharmacol. Transl. Sci..

[12] Robinson G.I., Li D., Wang B., Zahoruiko Y., Gerasymchuk M., Hudson D., Kovalchuk O., Kovalchuk I. (2023). Anti-Inflammatory Effects of Serotonin Receptor and Transient Receptor Potential Channel Ligands in Human Small Intestinal Epithelial Cells. Curr. Issues Mol. Biol..

[13] Rapalli A., Bertoni S., Arcaro V., Saccani F., Grandi A., Vivo V., Cantoni A.M., Barocelli E. (2016). Dual Role of Endogenous Serotonin in 2,4,6-Trinitrobenzene Sulfonic Acid-Induced Colitis. Front. Pharmacol..

[14] Xiao J., Shao L., Shen J., Jiang W., Feng Y., Zheng P., Liu F. (2016). Effects of ketanserin on experimental colitis in mice and macrophage function. Int. J. Mol. Med..

[15] Tang Q., Li Z.-Q., Li W., Guo J., Sun H.-Y., Zhang X.-H., Lau C.-P., Tse H.-F., Zhang S., Li G.-R. (2008). The 5-HT2 antagonist ketanserin is an open channel blocker of human cardiac ether-à-go-go-related gene (hERG) potassium channels. Br. J. Pharmacol..

[16] Aronson J.K. (2016). Ketanserin. Meyler’s Side Effects of Drugs (Sixteenth Edition).

[17] Pope J., Fenlon D., Thompson A., Shea B., Furst D., Wells G.A., Silman A. (1998). Ketanserin for Raynaud’s phenomenon in progressive systemic sclerosis. Cochrane Database Syst. Rev..

[18] Wu J., Li Z., Deng Y., Lu X., Luo C., Mu X., Zhang T., Liu Q., Tang S., Li J. (2023). Function of TRP channels in monocytes/macrophages. Front. Immunol..

[19] Parenti A., De Logu F., Geppetti P., Benemei S. (2016). What is the evidence for the role of TRP channels in inflammatory and immune cells?. Br. J. Pharmacol..

[20] Horikawa R., Oe Y., Fujii R., Kasuga R., Yoshimura R., Miyata S. (2022). Effects of peripheral administration of lipopolysaccharide on chronic sickness responses in TRPM8-deficient mice. Neurosci. Lett..

[21] Piciu F., Balas M., Badea M.A., Cucu D. (2023). TRP Channels in Tumoral Processes Mediated by Oxidative Stress and Inflammation. Antioxidants.

[22] Zanikov T., Gerasymchuk M., Ghasemi Gojani E., Robinson G.I., Asghari S., Groves A., Haselhorst L., Nandakumar S., Stahl C., Cameron M. (2023). The Effect of Combined Treatment of Psilocybin and Eugenol on Lipopolysaccharide-Induced Brain Inflammation in Mice. Molecules.

[23] Nisar M.F., Khadim M., Rafiq M., Chen J., Yang Y., Wan C.C. (2021). Pharmacological Properties and Health Benefits of Eugenol: A Comprehensive Review. Oxid. Med. Cell. Longev..

[24] Zhang J., Zheng Y., Luo Y., Du Y., Zhang X., Fu J. (2019). Curcumin inhibits LPS-induced neuroinflammation by promoting microglial M2 polarization via TREM2/TLR4/NF-κB pathways in BV2 cells. Mol. Immunol..

[25] Karthikeyan A., Young K.N., Moniruzzaman M., Beyene A.M., Do K., Kalaiselvi S., Min T. (2021). Curcumin and Its Modified Formulations on Inflammatory Bowel Disease (IBD): The Story So Far and Future Outlook. Pharmaceutics.

[26] EFSA (2010). Panel on Food Additives and Nutrient Sources added to Food (ANS) Scientific Opinion on the re-evaluation of curcumin (E 100) as a food additive. EFSA J..

[27] Csekő K., Beckers B., Keszthelyi D., Helyes Z. (2019). Role of TRPV1 and TRPA1 Ion Channels in Inflammatory Bowel Diseases: Potential Therapeutic Targets?. Pharmaceutics.

[28] Dos Santos E.A., Alvarez-Leite J.I. (2019). Capsaicin: A Potential Therapy Adjuvant for Intestinal Bowel Disease. J. Dig. Disord. Diagn..

[29] Zhang Q., Luo P., Xia F., Tang H., Chen J., Zhang J., Liu D., Zhu Y., Liu Y., Gu L. (2022). Capsaicin ameliorates inflammation in a TRPV1-independent mechanism by inhibiting PKM2-LDHA-mediated Warburg effect in sepsis. Cell Chem. Biol..

[30] Kang C., Wang B., Kaliannan K., Wang X., Lang H., Hui S., Huang L., Zhang Y., Zhou M., Chen M. (2017). Gut Microbiota Mediates the Protective Effects of Dietary Capsaicin against Chronic Low-Grade Inflammation and Associated Obesity Induced by High-Fat Diet. mBio.

[31] Arnold J.T., Stewart S.B.-L., Sammut L. (2018). Oral Capsaicin Ingestion: A Brief Update Dose, Tolerance and Side Effects. Res. Rev. J. Herb. Sci..

[32] Berg K.A., Maayani S., Goldfarb J., Clarke W.P. (1998). Pleiotropic behavior of 5-HT2A and 5-HT2C receptor agonists. Ann. N. Y. Acad. Sci..

[33] Markus J., Landry T., Stevens Z., Scott H., Llanos P., Debatis M., Armento A., Klausner M., Ayehunie S. (2021). Human small intestinal organotypic culture model for drug permeation, inflammation, and toxicity assays. In Vitro Cell. Dev. Biol. Anim..

[34] Kovalchuk A., Wang B., Li D., Rodriguez-Juarez R., Ilnytskyy S., Kovalchuk I., Kovalchuk O. (2021). Fighting the storm: Could novel anti-TNFα and anti-IL-6 C. sativa cultivars tame cytokine storm in COVID-19?. Aging.

[35] Woznicki J.A., Saini N., Flood P., Rajaram S., Lee C.M., Stamou P., Skowyra A., Bustamante-Garrido M., Regazzoni K., Crawford N. (2021). TNF-α synergises with IFN-γ to induce caspase-8-JAK1/2-STAT1-dependent death of intestinal epithelial cells. Cell Death Dis..

[36] Chen C. (2010). COX-2′s new role in inflammation. Nat. Chem. Biol..

[37] Ricciotti E., FitzGerald G.A. (2011). Prostaglandins and Inflammation. Arterioscler. Thromb. Vasc. Biol..

[38] Kontoyiannis D., Pasparakis M., Pizarro T.T., Cominelli F., Kollias G. (1999). Impaired On/Off Regulation of TNF Biosynthesis in Mice Lacking TNF AU-Rich Elements: Implications for Joint and Gut-Associated Immunopathologies. Immunity.

[39] Schmitt H., Billmeier U., Dieterich W., Rath T., Sonnewald S., Reid S., Hirschmann S., Hildner K., Waldner M.J., Mudter J. (2019). Expansion of IL-23 receptor bearing TNFR2+ T cells is associated with molecular resistance to anti-TNF therapy in Crohn’s disease. Gut.

[40] Ivashkiv L.B. (2018). IFNγ: Signalling, epigenetics and roles in immunity, metabolism, disease and cancer immunotherapy. Nat. Rev. Immunol..

[41] Thapa R.J., Basagoudanavar S.H., Nogusa S., Irrinki K., Mallilankaraman K., Slifker M.J., Beg A.A., Madesh M., Balachandran S. (2011). NF-kappaB protects cells from gamma interferon-induced RIP1-dependent necroptosis. Mol. Cell. Biol..

[42] Thapa R.J., Nogusa S., Chen P., Maki J.L., Lerro A., Andrake M., Rall G.F., Degterev A., Balachandran S. (2013). Interferon-induced RIP1/RIP3-mediated necrosis requires PKR and is licensed by FADD and caspases. Proc. Natl. Acad. Sci. USA.

[43] Ingram J.P., Thapa R.J., Fisher A., Tummers B., Zhang T., Yin C., Rodriguez D.A., Guo H., Lane R., Williams R. (2019). ZBP1/DAI Drives RIPK3-Mediated Cell Death Induced by IFNs in the Absence of RIPK1. J. Immunol..

[44] Fuss I.J., Neurath M., Boirivant M., Klein J.S., de la Motte C., Strong S.A., Fiocchi C., Strober W. (1996). Disparate CD_4_^+^ lamina propria (LP) lymphokine secretion profiles in inflammatory bowel disease. Crohn’s disease LP cells manifest increased secretion of IFN-gamma, whereas ulcerative colitis LP cells manifest increased secretion of IL-5. J. Immunol..

[45] Breese E., Braegger C.P., Corrigan C.J., Walker-Smith J.A., MacDonald T.T. (1993). Interleukin-2- and interferon-gamma-secreting T cells in normal and diseased human intestinal mucosa. Immunology.

[46] Camoglio L., Te Velde A.A., Tigges A.J., Das P.K., Van Deventer S.J. (1998). Altered expression of interferon-gamma and interleukin-4 in inflammatory bowel disease. Inflamm. Bowel Dis..

[47] Deem R.L., Shanahan F., Targan S.R. (1991). Triggered human mucosal T cells release tumour necrosis factor-alpha and interferon-gamma which kill human colonic epithelial cells. Clin. Exp. Immunol..

[48] Bruewer M., Luegering A., Kucharzik T., Parkos C.A., Madara J.L., Hopkins A.M., Nusrat A. (2003). Proinflammatory cytokines disrupt epithelial barrier function by apoptosis-independent mechanisms. J. Immunol..

[49] Mitsuyama K., Toyonaga A., Sasaki E., Ishida O., Ikeda H., Tsuruta O., Harada K., Tateishi H., Nishiyama T., Tanikawa K. (1995). Soluble interleukin-6 receptors in inflammatory bowel disease: Relation to circulating interleukin-6. Gut.

[50] Atreya R., Mudter J., Finotto S., Müllberg J., Jostock T., Wirtz S., Schütz M., Bartsch B., Holtmann M., Becker C. (2000). Blockade of interleukin 6 trans signaling suppresses T-cell resistance against apoptosis in chronic intestinal inflammation: Evidence in Crohn disease and experimental colitis in vivo. Nat. Med..

[51] Ghandadi M., Sahebkar A. (2017). Curcumin: An Effective Inhibitor of Interleukin-6. Curr. Pharm. Des..

[52] Gorabi A.M., Razi B., Aslani S., Abbasifard M., Imani D., Sathyapalan T., Sahebkar A. (2021). Effect of curcumin on proinflammatory cytokines: A meta-analysis of randomized controlled trials. Cytokine.

[53] Takada Y., Hisamatsu T., Kamada N., Kitazume M.T., Honda H., Oshima Y., Saito R., Takayama T., Kobayashi T., Chinen H. (2010). Monocyte Chemoattractant Protein-1 Contributes to Gut Homeostasis and Intestinal Inflammation by Composition of IL-10–Producing Regulatory Macrophage Subset. J. Immunol..

[54] Lim J.H., Kwon T.K. (2010). Curcumin inhibits phorbol myristate acetate (PMA)-induced MCP-1 expression by inhibiting ERK and NF-kappaB transcriptional activity. Food Chem. Toxicol. Int. J. Publ. Br. Ind. Biol. Res. Assoc..

[55] Karimian M.S., Pirro M., Majeed M., Sahebkar A. (2017). Curcumin as a natural regulator of monocyte chemoattractant protein-1. Cytokine Growth Factor Rev..

[56] Ariki S., Ozaka S., Sachi N., Chalalai T., Soga Y., Fukuda C., Kagoshima Y., Ekronarongchai S., Mizukami K., Kamiyama N. (2023). GM-CSF-producing CCR2+ CCR6+ Th17 cells are pathogenic in dextran sodium sulfate-induced colitis model in mice. Genes Cells.

[57] Ebbo M., Crinier A., Vély F., Vivier E. (2017). Innate lymphoid cells: Major players in inflammatory diseases. Nat. Rev. Immunol..

[58] Önal H.T., Yetkin D., Ayaz F. (2023). Immunostimulatory activity of fluoxetine in macrophages via regulation of the PI3K and P38 signaling pathways. Immunol. Res..

[59] Zhao X., Dong B., Friesen M., Liu S., Zhu C., Yang C. (2021). Capsaicin Attenuates Lipopolysaccharide-Induced Inflammation and Barrier Dysfunction in Intestinal Porcine Epithelial Cell Line-J2. Front. Physiol..

[60] Azimirad M., Noori M., Azimirad F., Gholami F., Naseri K., Yadegar A., Asadzadeh Aghdaei H., Zali M.R. (2022). Curcumin and capsaicin regulate apoptosis and alleviate intestinal inflammation induced by Clostridioides difficile in vitro. Ann. Clin. Microbiol. Antimicrob..

[61] Cione E., Plastina P., Pingitore A., Perri M., Caroleo M.C., Fazio A., Witkamp R., Meijerink J. (2019). Capsaicin Analogues Derived from n-3 Polyunsaturated Fatty Acids (PUFAs) Reduce Inflammatory Activity of Macrophages and Stimulate Insulin Secretion by β-Cells In Vitro. Nutrients.

[62] Kunde D.A., Yingchoncharoen J., Jurković S., Geraghty D.P. (2018). TRPV1 mediates capsaicin-stimulated metabolic activity but not cell death or inhibition of interleukin-1β release in human THP-1 monocytes. Toxicol. Appl. Pharmacol..

[63] Calzetta L., Pistocchini E., Cito G., Ritondo B.L., Verri S., Rogliani P. (2022). Inflammatory and contractile profile in LPS-challenged equine isolated bronchi: Evidence for IL-6 as a potential target against AHR in equine asthma. Pulm. Pharmacol. Ther..

[64] Rodriguiz R.M., Nadkarni V., Means C.R., Pogorelov V.M., Chiu Y.-T., Roth B.L., Wetsel W.C. (2021). LSD-stimulated behaviors in mice require β-arrestin 2 but not β-arrestin 1. Sci. Rep..

[65] Sharma D., Parameswaran N. (2015). Multifaceted role of β-arrestins in inflammation and disease. Genes Immun..

[66] Cheshmehkani A., Senatorov I.S., Dhuguru J., Ghoneim O., Moniri N.H. (2017). Free-fatty acid receptor-4 (FFA4) modulates ROS generation and COX-2 expression via the C-terminal β-arrestin phosphosensor in Raw 264.7 macrophages. Biochem. Pharmacol..

[67] Huang T., Chen X., Chen D., Yu B., He J., Yan H., Luo Y., Zheng P., Chen H., Huang Z. (2023). Eugenol promotes appetite through TRP channels mediated-CaMKK2/AMPK signaling pathway. Phytother. Res..

[68] Racioppi L., Nelson E.R., Huang W., Mukherjee D., Lawrence S.A., Lento W., Masci A.M., Jiao Y., Park S., York B. (2019). CaMKK2 in myeloid cells is a key regulator of the immune-suppressive microenvironment in breast cancer. Nat. Commun..

[69] Fedi A., Vitale C., Ponschin G., Ayehunie S., Fato M., Scaglione S. (2021). In vitro models replicating the human intestinal epithelium for absorption and metabolism studies: A systematic review. J. Control. Release.

[70] Cao D., Yu J., Wang H., Luo Z., Liu X., He L., Qi J., Fan L., Tang L., Chen Z. (2022). Structure-based discovery of nonhallucinogenic psychedelic analogs. Science.

[71] González-Maeso J., Weisstaub N.V., Zhou M., Chan P., Ivic L., Ang R., Lira A., Bradley-Moore M., Ge Y., Zhou Q. (2007). Hallucinogens recruit specific cortical 5-HT(2A) receptor-mediated signaling pathways to affect behavior. Neuron.

[72] Liu C., Zhang X., Zhou J.-X., Wei W., Liu D.-H., Ke P., Zhang G.-F., Cai G.-J., Su D.-F. (2013). The protective action of ketanserin against lipopolysaccharide-induced shock in mice is mediated by inhibiting inducible NO synthase expression via the MEK/ERK pathway. Free Radic. Biol. Med..

[73] Sengupta T.K., Talbot E.S., Scherle P.A., Ivashkiv L.B. (1998). Rapid inhibition of interleukin-6 signaling and Stat3 activation mediated by mitogen-activated protein kinases. Proc. Natl. Acad. Sci. USA.

[74] Seidel M.F., Fiebich B.L., Ulrich-Merzenich G., Candelario-Jalil E., Koch F.-W., Vetter H. (2008). Serotonin mediates PGE2 overexpression through 5-HT2A and 5-HT3 receptor subtypes in serum-free tissue culture of macrophage-like synovial cells. Rheumatol. Int..

[75] Cloëz-Tayarani I., Petit-Bertron A.-F., Venters H.D., Cavaillon J.-M. (2003). Differential effect of serotonin on cytokine production in lipopolysaccharide-stimulated human peripheral blood mononuclear cells: Involvement of 5-hydroxytryptamine2A receptors. Int. Immunol..

[76] Yang M., Srikiatkhachorn A., Anthony M., Chong B.H. (1996). Serotonin stimulates megakaryocytopoiesis via the 5-HT2 receptor. Blood Coagul. Fibrinolysis.

[77] Sviridova A., Rogovskii V., Kudrin V., Pashenkov M., Boyko A., Melnikov M. (2021). The role of 5-HT2B-receptors in fluoxetine-mediated modulation of Th17- and Th1-cells in multiple sclerosis. J. Neuroimmunol..

[78] Tylš F., Páleníček T., Horáček J. (2014). Psilocybin—Summary of knowledge and new perspectives. Eur. Neuropsychopharmacol..

[79] Jones N.T., Zahid Z., Grady S.M., Sultan Z.W., Zheng Z., Razidlo J., Banks M.I., Wenthur C.J. (2023). Transient Elevation of Plasma Glucocorticoids Supports Psilocybin-Induced Anxiolysis in Mice. ACS Pharmacol. Transl. Sci..

[80] Liu N., Sun S., Wang P., Sun Y., Hu Q., Wang X. (2021). The Mechanism of Secretion and Metabolism of Gut-Derived 5-Hydroxytryptamine. Int. J. Mol. Sci..

[81] Bischoff S.C., Barbara G., Buurman W., Ockhuizen T., Schulzke J.-D., Serino M., Tilg H., Watson A., Wells J.M. (2014). Intestinal permeability—A new target for disease prevention and therapy. BMC Gastroenterol..

[82] Wang B., Sun S., Liu M., Chen H., Liu N., Wu Z., Wu G., Dai Z. (2020). Dietary L-Tryptophan Regulates Colonic Serotonin Homeostasis in Mice with Dextran Sodium Sulfate-Induced Colitis. J. Nutr..

[83] Koopman N., Katsavelis D., ten Hove A.S., Brul S., de Jonge W.J., Seppen J. (2021). The Multifaceted Role of Serotonin in Intestinal Homeostasis. Int. J. Mol. Sci..

[84] Nau F., Yu B., Martin D., Nichols C.D. (2013). Serotonin 5-HT2A Receptor Activation Blocks TNF-α Mediated Inflammation In Vivo. PLoS ONE.

[85] Mao L., Xin F., Ren J., Xu S., Huang H., Zha X., Wen X., Gu G., Yang G., Cheng Y. (2022). 5-HT2B-mediated serotonin activation in enterocytes suppresses colitis-associated cancer initiation and promotes cancer progression. Theranostics.

[86] Lee S.H., eun Kwon J., Cho M.-L. (2018). Immunological pathogenesis of inflammatory bowel disease. Intest. Res..

[87] Zinberg N.E. (1986). Drug, Set, and Setting: The Basis for Controlled Intoxicant Use.

[88] Schlag A.K., Aday J., Salam I., Neill J.C., Nutt D.J. (2022). Adverse effects of psychedelics: From anecdotes and misinformation to systematic science. J. Psychopharmacol. Oxf. Engl..

[89] Irizarry R., Winczura A., Dimassi O., Dhillon N., Minhas A., Larice J. (2022). Psilocybin as a Treatment for Psychiatric Illness: A Meta-Analysis. Cureus.

[90] Ona G., Bouso J.C. (2020). Potential safety, benefits, and influence of the placebo effect in microdosing psychedelic drugs: A systematic review. Neurosci. Biobehav. Rev..

[91] Kuypers K.P.C. (2020). The therapeutic potential of microdosing psychedelics in depression. Ther. Adv. Psychopharmacol..

[92] Mortensen J.K., Andersen G. (2022). Safety considerations for prescribing SSRI antidepressants to patients at increased cardiovascular risk. Expert Opin. Drug Saf..

[93] Ledwos N., Rosenblat J.D., Blumberger D.M., Castle D.J., McIntyre R.S., Mulsant B.H., Husain M.I. (2022). A Critical Appraisal of Evidence on the Efficacy and Safety of Serotonergic Psychedelic Drugs as Emerging Antidepressants: Mind the Evidence Gap. J. Clin. Psychopharmacol..

[94] Rosenblat J.D., Husain M.I., Lee Y., McIntyre R.S., Mansur R.B., Castle D., Offman H., Parikh S.V., Frey B.N., Schaffer A. (2023). The Canadian Network for Mood and Anxiety Treatments (CANMAT) Task Force Report: Serotonergic Psychedelic Treatments for Major Depressive Disorder. Can. J. Psychiatry.

[95] Hernandez-Leon A., Escamilla-Orozco R.I., Tabal-Robles A.R., Martínez-Vargas D., Romero-Bautista L., Escamilla-Soto G., González-Romero O.S., Torres-Valencia M., González-Trujano M.E. (2024). Antidepressant- and anxiolytic-like activities and acute toxicity evaluation of the Psilocybe cubensis mushroom in experimental models in mice. J. Ethnopharmacol..

[96] Lam N.T., Balachandran K. (2015). The mechanobiology of drug-induced cardiac valve disease. J. Long. Term Eff. Med. Implants.

[97] McIntyre R.S. (2023). Serotonin 5-HT2B receptor agonism and valvular heart disease: Implications for the development of psilocybin and related agents. Expert Opin. Drug Saf..

[98] Rickli A., Moning O.D., Hoener M.C., Liechti M.E. (2016). Receptor interaction profiles of novel psychoactive tryptamines compared with classic hallucinogens. Eur. Neuropsychopharmacol..

[99] Glatfelter G.C., Pham D.N.K., Walther D., Golen J.A., Chadeayne A.R., Baumann M.H., Manke D.R. (2022). Synthesis, Structural Characterization, and Pharmacological Activity of Novel Quaternary Salts of 4-Substituted Tryptamines. ACS Omega.

[100] Dahmane E., Hutson P.R., Gobburu J.V.S. (2021). Exposure-Response Analysis to Assess the Concentration-QTc Relationship of Psilocybin/Psilocin. Clin. Pharmacol. Drug Dev..

[101] Mawe G.M., Hoffman J.M. (2013). Serotonin Signaling in the Gastrointestinal Tract: *Nat*. Rev. Gastroenterol. Hepatol..

[102] Fiorica-Howells E., Hen R., Gingrich J., Li Z., Gershon M.D. (2002). 5-HT2A receptors: Location and functional analysis in intestines of wild-type and 5-HT2A knockout mice. Am. J. Physiol.-Gastrointest. Liver Physiol..

[103] Panpetch W., Visitchanakun P., Saisorn W., Sawatpanich A., Chatthanathon P., Somboonna N., Tumwasorn S., Leelahavanichkul A. (2021). Lactobacillus rhamnosus attenuates Thai chili extracts induced gut inflammation and dysbiosis despite capsaicin bactericidal effect against the probiotics, a possible toxicity of high dose capsaicin. PLoS ONE.

[104] Mohammadi A., Blesso C.N., Barreto G.E., Banach M., Majeed M., Sahebkar A. (2019). Macrophage plasticity, polarization and function in response to curcumin, a diet-derived polyphenol, as an immunomodulatory agent. J. Nutr. Biochem..

