# Supplementary material for: Psilocybin and Eugenol Reduce Inflammation in Human 3D EpiIntestinal Tissue
Source: Life (Basel). 2023 Dec 15;13(12):2345. doi: 10.3390/life13122345 (PMC10744792; doi:10.3390/life13122345)
Supplement: Supplementary file 1 [file life-13-02345-s001.zip › life-2615100-supplementary.pdf]

Supplementary Materials

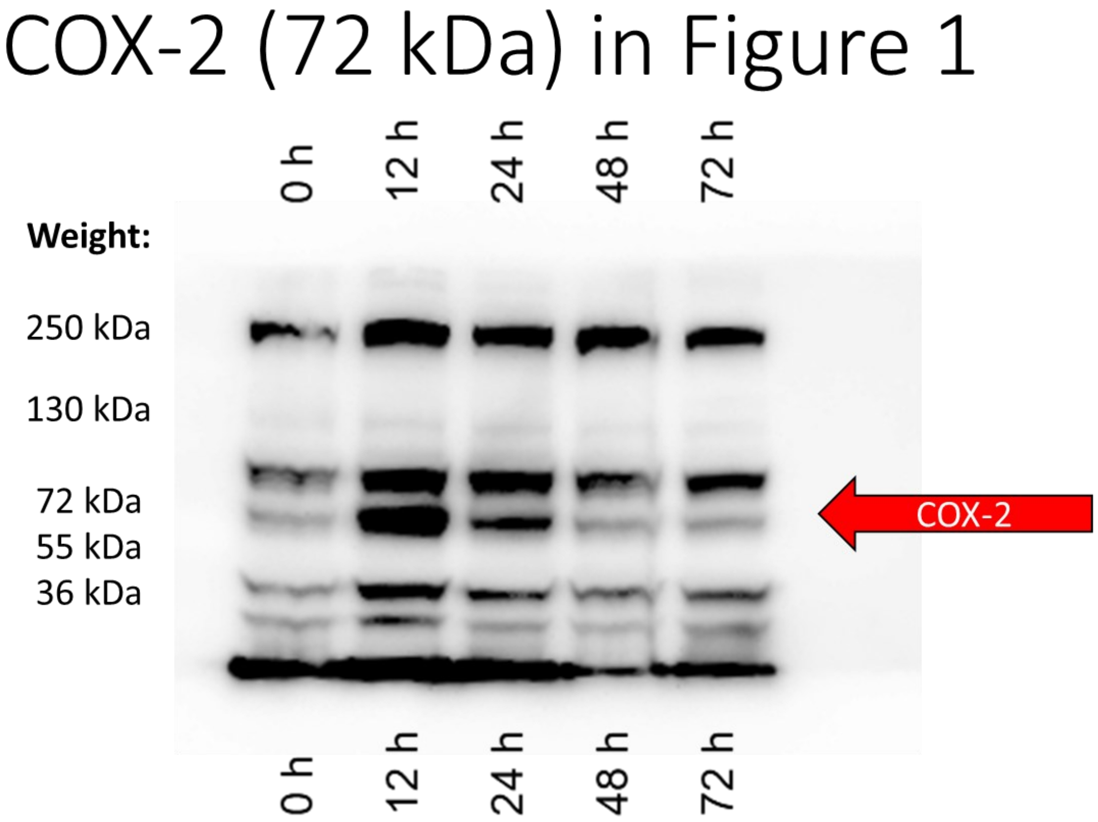

Figure S1. Original Western blots of proteins from human 3D EpiIntestinal tissue showing COX-2 at molecular weight of 72 kDa. Red arrow indicates bands shown in Figure 1B.

GAPDH (36 kDa) in Figure 1

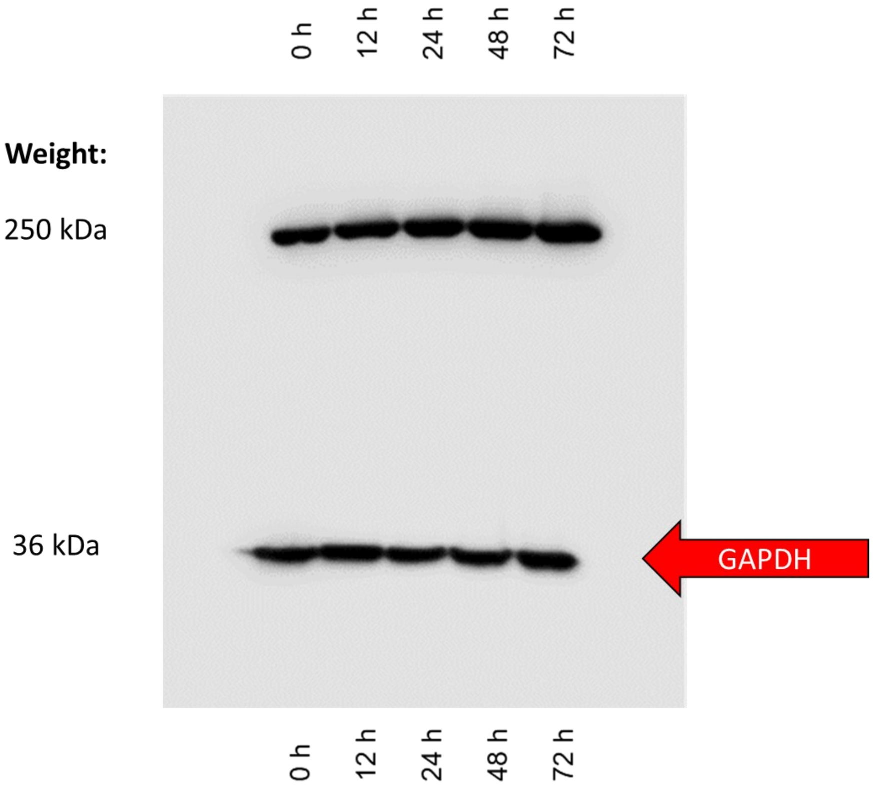

Figure S2. Original Western blots of proteins from human 3D EpiIntestinal tissue showing GAPDH at molecular weight of 36 kDa. Red arrow indicates bands shown in Figure 1B.
